# Genetic Background but Not Intestinal Microbiota After Co-Housing Determines Hyperoxaluria-Related Nephrocalcinosis in Common Inbred Mouse Strains

**DOI:** 10.3389/fimmu.2021.673423

**Published:** 2021-04-21

**Authors:** Qiuyue Ma, Melissa Grigorescu, Adrian Schreiber, Ralph Kettritz, Maja Lindenmeyer, Hans-Joachim Anders, Stefanie Steiger

**Affiliations:** ^1^ Division of Nephrology, Department of Medicine IV, University Hospital, Ludwig-Maximilians-University Munich, Munich, Germany; ^2^ Department of Nephrology and Medical Intensive Care, Charité-Universitätsmedizin Berlin, Berlin, Germany; ^3^ Experimental and Clinical Research Center, Charité-Universitätsmedizin Berlin and Max Delbrueck Center for Molecular Medicine in the Helmholtz Association, Berlin, Germany; ^4^ III. Department of Medicine University Medical Center Hamburg-Eppendorf, Hamburg, Germany

**Keywords:** calcium oxalate, nephrocalcinosis, microbiota, mouse strains, uromodulin, kidney stone disease

## Abstract

Calcium oxalate (CaOx) crystal formation, aggregation and growth is a common cause of kidney stone disease and nephrocalcinosis-related chronic kidney disease (CKD). Genetically modified mouse strains are frequently used as an experimental tool in this context but observed phenotypes may also relate to the genetic background or intestinal microbiota. We hypothesized that the genetic background or intestinal microbiota of mice determine CaOx crystal deposition and thus the outcome of nephrocalcinosis. Indeed, *Casp1* ^-/-^, *Cybb* ^-/-^ or *Casp1* ^-/-^/*Cybb* ^-/-^ knockout mice on a 129/C57BL/6J (B6J) background that were fed an oxalate-rich diet for 14 days did neither encounter intrarenal CaOx crystal deposits nor nephrocalcinosis-related CKD. To test our assumption, we fed C57BL/6N (B6N), 129, B6J and Balb/c mice an oxalate-rich diet for 14 days. Only B6N mice displayed CaOx crystal deposits and developed CKD associated with tubular injury, inflammation and interstitial fibrosis. Intrarenal mRNA expression profiling of 64 known nephrocalcinosis-related genes revealed that healthy B6N mice had lower mRNA levels of uromodulin (*Umod*) compared to the other three strains. Feeding an oxalate-rich diet caused an increase in uromodulin protein expression and CaOx crystal deposition in the kidney as well as in urinary uromodulin excretion in B6N mice but not 129, B6J and Balb/c mice. However, backcrossing 129 mice on a B6N background resulted in a gradual increase in CaOx crystal deposits from F2 to F7, of which all B6N/129 mice from the 7^th^ generation developed CaOx-related nephropathy similar to B6N mice. Co-housing experiments tested for a putative role of the intestinal microbiota but B6N co-housed with 129 mice or B6N/129 (3^rd^ and 6^th^ generation) mice did not affect nephrocalcinosis. In summary, genetic background but not the intestinal microbiome account for strain-specific crystal formation and, the levels of uromodulin secretion may contribute to this phenomenon. Our results imply that only littermate controls of the identical genetic background strain are appropriate when performing knockout mouse studies in this context, while co-housing is optional.

## Introduction

Calcium oxalate crystals is the main constituent of most kidney stones and account for approximately 75% of crystal-related kidney damage and eventually kidney failure (1). Hyperoxaluria-related CaOx stones form when the urine becomes excessively supersaturated due to the intake of oxalate-rich food, leading to crystal formation, growth, aggregation and retention in the renal tubular lumen (2). This process is independent of urinary pH (3) but affected by the composition of minerals or proteins, such as calcium, phosphate, magnesium, citrate, uromodulin, nephrocalcin, osteopontin, calgranulin and macroglobulin (4). Risk factors, such as oxalate-rich diet, obesity, diabetes, hypertension and metabolic syndrome, have been linked to contribute to CaOx stone formation (5). Such CaOx crystals can cause an inflammatory response associated with the release of pro-inflammatory mediators, cell death and leukocyte infiltration, which further contribute to tubular atrophy and interstitial fibrosis, leading to progressive nephrocalcinosis (6). A better understanding of the mechanisms involved in CaOx stones formation to predict and facilitate the development of more-effective drugs are of great need for preventing and treating this disease.

Epidemiological studies reveal that genetic factors play a pivotal role in kidney stone formation (7, 8). To study the pathogenesis of different types of kidney stones or crystal-induced chronic kidney disease (9, 10), mice are most commonly used as experimental models. Although studies using genetically modified mouse strains indicate an association of certain genes with CaOx crystal formation (11–13), the role of the genetic predisposition remains poorly defined. Moreover, experimentally induced hyperoxaluria may not always lead to CaOx crystal formation (4). Inbred mouse strains, especially C57BL/6 mice including the B6N and B6J substrains are often used to study the pathogenesis of diseases despite the genetic variants that can cause phenotypic differences (14).

Genetically modified mice are usually generated using germline transmission competent embryonic stem (ES) cell lines derived from the 129 mouse strain (15, 16). 129 ES cell lines are easy to handle in culture and remain competent to repopulate the mouse germline. However, 129 chimeric mouse strains do not breed well and can possess anatomical and behavioral abnormalities (17). For phenotypic studies, 129 chimeric mice are backcrossed repeatedly to a particular strain, often C57BL/6, whereby the genomic signature of the congenic mice (129/B6) depends on the number of backcrosses. Backcrossing for more than 10 generations is desirable to increase the genetic homogeneity (18) but to decrease the impact of passenger mutations that affect phenotypes (16), genetic matters critical for the validation and reproducibility of experimental studies (19).

Clinical evidence suggests a strong association between the intestinal microbiota and kidney stone disease (20). These studies found that the intestinal microbiome of patients suffering from kidney stones is less diverse than in individuals without a history of stones (21, 22), and that certain bacteria including *Oxalobacter* spp., which degrade oxalate, were less abundant in the stool of adults with kidney stones (23, 24). Such imbalances in the microbiota contribute to epithelial barrier dysfunction and alterations in the immune response (25). In addition, the frequent use of antibiotics may modify the intestinal microbiome; thus, increasing the risk for stone formation in children and young adults (26, 27).

In this study, we investigated whether the genetic background of mice affects CaOx crystal deposition in the kidneys and consequently the outcomes of CaOx-related nephrocalcinosis in four different inbred mouse strains and whether co-housing for alignment of the intestinal microbiome might contribute to these physiological processes.

## Materials and Methods

### Animal Studies

Seven or eight-week old common inbred C57BL/6N (B6N), C57BL/6J (B6J), 129/Sv (129) and Balb/c mouse strains (male) were purchased from Charles River Laboratories (Sulzfeld, Germany). *Casp1* ^-/-^, *Cybb* ^-/-^ and *Casp1*/*Cybb* ^-/-^ mice (generated in 129-derived ES cells) on a B6J background (28). For backcrossing studies, 129/B6N mice were generated by breeding 129 mice with B6N mice for up to 7 generations (F2, F3, F5, F6 and F7). Black male mice were used for experiments. For co-housing experiments, 3 week-old male B6N mice were co-housed with 3 week-old male 129 inbred and 129/B6N F3 or F6 mice for 4 weeks prior to induction of kidney disease, a tool to standardize the intestinal microbiota in animals (29, 30). Mice were housed in groups of five in filter top cages under a 12-hour light/dark cycle environment with unlimited access to food and water. Cages, nest lets, food, and water were sterilized by autoclaving before use. Group size calculation for the primary endpoint was based on numeric assumptions derived from our previous experience with this animal model (31–34). Oxalate-rich diet was prepared by adding sodium oxalate (50 μmol/g) to a calcium-free diet or calcium-free diet without sodium oxalate (control diet, both from Ssniff, Soest, Germany) (31–33). Mice were placed either on an oxalate-rich diet or control diet for 14 days. Plasma and urine samples were collected on day 0 and before sacrifice on day 14, and stored at -20°C until analysis. Kidneys were harvested after sacrifice. One kidney was kept in 4% formalin to be embedded in paraffin for histology analysis and the second kidney placed in RNAlater solution (Qiagen, Hilden, Germany) at -80°C for qPCR analyses.

### Primary End Point

#### Pizzolato staining

Kidney sections of 2 μm were used for immunostaining. To visualize CaOx crystals, kidney sections were stained by Pizzolato and the percentage (%) area of crystal deposits in the kidney quantified using ImageJ software (35, 36). An observer blinded to the experimental condition performed all assessments.

### Secondary Analyses

#### Assessment of Plasma BUN and Creatinine

Plasma blood urea nitrogen (BUN) and creatinine (both from DiaSys, Holzheim, Germany) levels were measured using commercially available kits as per manufacturer’s protocol.

#### Assessment of Kidney Histology

The expression of crystal adhesion molecules CD44 and annexin II were identified by immunostaining for CD44 and annexin II (both from Abcam, Inc., Cambridge, MA). Quantification of immunostaining (% area) was done using ImageJ software. For assessment of kidney injury and interstitial fibrosis, we stained 2-µm thick kidney sections with periodic acid-Schiff (PAS) reagent and silver stain, respectively. Kidney injury was scored by assessing the percentage of necrotic tubules, dilation and casts (33). Kidney sections were stained for Tamm-Horsfall protein (THP) to illustrate uromodulin protein expression. All assessments were performed by an observer blinded to the experimental conditions.

#### Measurement of Urinary pH, Oxalate, Calcium and Uromodulin

Fresh urine pH was measured using a pH meter (SenTix, Germany). Afterward, urine samples were acidified to determine urinary oxalic acid concentrations using a colorimetric enzymatic assay (Oxalate assay kit, Libios) in 96-well plates according to the manufacturer’s instructions. Urine calcium concentrations were assessed using the calcium colorimetric assay kit (Sigma-Aldrich, Fell, Germany) and urine uromodulin levels using the mouse uromodulin ELISA kit (MyBioSource, Germany).

#### RNA Preparation and Real-Time Quantitative–PCR

Total RNA was isolated and purified from murine kidneys using a Qiagen RNA extraction kit (D̈sseldorf, Germany) according to the manufacturer’s instructions. RNA quality was assessed using agarose gels before being transcribed into cDNA using reverse transcriptase (Superscript II, Invitrogen, Carlsbad, CA). Real-time RT-PCR was performed using SYBR Green PCR master mix and analyzed with a Light Cycler 480 (Roche, Germany). All gene expression values (ct values) were normalized using *18s* rRNA as a housekeeping gene. All primers used for amplification were purchased from Metabion (Martinsried, Germany) and are listed in **Supplementary Table S1**.

### Gene Expression Analysis

We analyzed gene expression patterns in the liver and kidney of healthy B6N, B6J and 129 mice. Published datasets of liver samples from healthy B6N, 129 and B6J mice were used for gene expression analysis (GSE43106) (37). CEL file >normalization was performed with the Robust Multichip Average method using RMAExpress (Version 1.0.5) and the mouse Entrez‐Gene custom CDF annotation from Brain Array version 20 (http://brainarray.mbni.med.umich.edu/Brainarray/Database/CustomCDF/CDF_download.asp). To identify differentially expressed genes, the SAM (Significance Analysis of Microarrays) method (38) was applied using the bioconductor package Sam.r. A q-value below 5% was considered to be statistically significant. 1211 genes in the BN6 vs 129 comparison and 5 genes in the B6N vs B6J comparison were significantly regulated (fold change cut-off of ≥2 or ≤0.5, q-value <5%). Of those liver genes we selected 229 liver genes with a fold change cut-off of ≥3 or ≤0.3 and q-value <5% for further analysis and found 21 genes (20 B6N vs 129 and 1 B6N vs B6J) highly expressed and 26 genes (24 B6N vs 129 and 2 B6N vs B6J) intermediately expressed in the kidney of healthy mice according to the NCBI gene database (https://www.ncbi.nlm.nih.gov/gene). Using RT-PCR, we found 15 genes that were differentially expressed in healthy kidneys between B6N vs 129 mice and B6N vs B6J mice.

### Calcium Oxalate Crystal Formation *In Vitro*

The formation of CaOx crystals *in vitro* has previously been described in more detail (35, 39). Briefly, urine from healthy B6N, 129, B6J and Balb/c mice was pre-incubated with or without 100 μl of a Na_2_C_2_O_4_ solution (oxalate, 0.1 mM, pH 7.3) for 1 hour at room temperature prior to incubation with 100 μl CaCl_2_ solution (0.1 mM, pH 7.3) for 5 minutes. CaOx crystals alone and urine alone served as controls. The formation of CaOx crystals was quantified by size (forward scatter versus sideward scatter) using the flow cytometer BD FACSCalibur (Becton Dickinson, NJ, USA).

### Statistical Analyses

Statistical analyses were performed using GraphPad Prism 7 (CA, USA). Data were normally distributed and compared by one-way analysis of variance (ANOVA) with Tukey’s post-test for three or more groups, or by two-way ANOVA with Bonferroni’s comparison post-hoc test when comparing two parameters with multiple groups. Data are presented as mean values ± standard deviation (SD). Differences were considered significant if p<0.05; no significant differences (ns) are indicated accordingly. Group sizes are indicated in each corresponding figure legend.

## Results

### Knockout Mice on a 129/B6J Background Do Not Develop CaOx-Related Nephrocalcinosis

**Figure 1:**
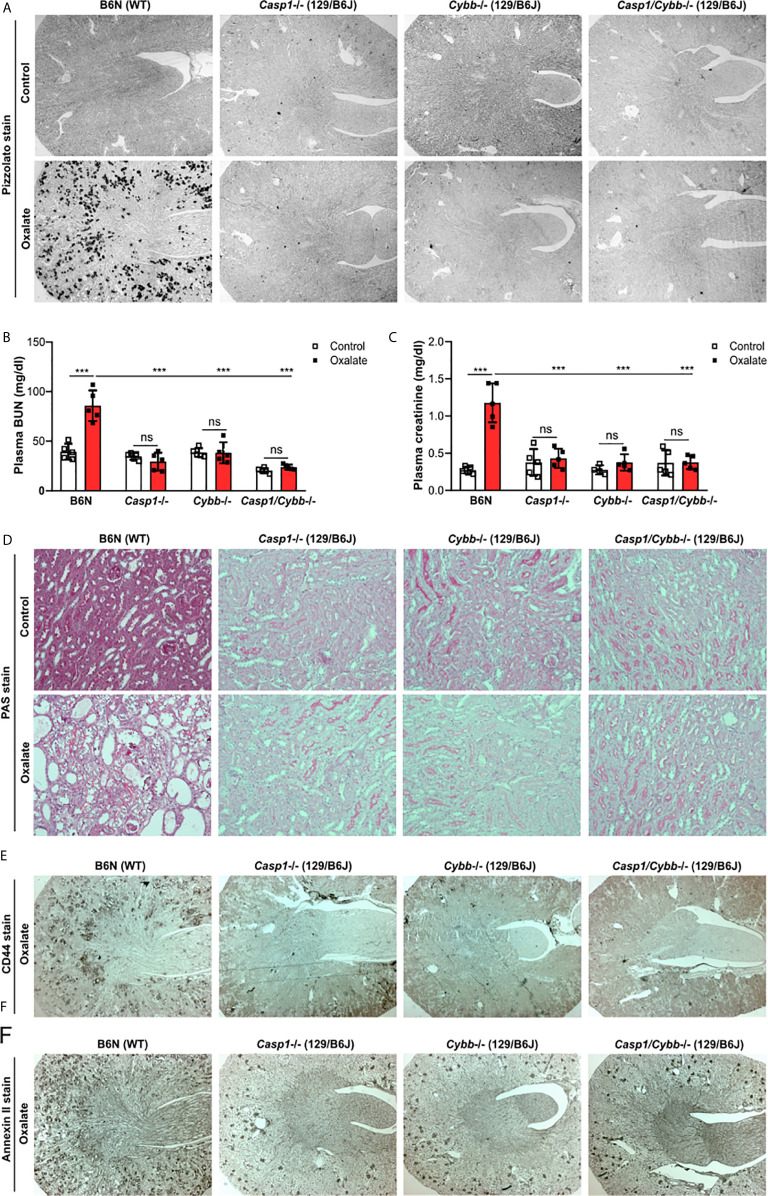
Knockout mice on a 129/B6J background do not develop CaOx-related nephrocalcinosis. C57BL/6N WT (B6N) and *Casp1*-, *Cybb*-, *Casp1/Cybb*- deficient (129/B6J background) mice were either fed a control diet or an oxalate-rich diet for 14 days. **(A)** Pizzolato staining to identify CaOx crystal deposits in kidney sections of all four mouse strains. **(B, C)** Plasma BUN **(B)** and plasma creatinine **(C)** levels were measured using colorimetric assays. **(D)** PAS stain illustrates tubular injury. Original magnification x200. **(E, F)** Immunostaining of the adhesion molecules CD44 **(E)** and annexin II **(F)** in kidney sections. Original magnification ×25. Data are mean ± SD from 4 to 5 mice per group. ***p < 0.001; ns, not significant by two-way ANOVA.

To address the potential contribution of caspase 1 and NADPH oxidase 2 ﻿during nephrocalcinosis, we fed 129/B6J mice deficient in *Casp1*, *Cybb* and *Casp1/Cybb* an oxalate-rich diet, a previously characterized mouse model of CaOx crystal-induced nephropathy (31). Feeding wild-type (WT) B6N mice an oxalate-rich diet triggered CaOx crystal formation in the medulla and cortex on day 14, as indicted by the Pizzolato staining of kidney sections, while CaOx crystal deposits were absent in *Casp1* ^-/-^, *Cybb* ^-/-^ and *Casp1*/*Cybb* ^-/-^ (129/B6J) mice (**Figure 1A**). The deposition of CaOx crystals resulted in an impaired kidney function in WT B6N mice, as indicated by significantly elevated plasma BUN and creatinine levels as compared to the control diet (**Figures 1B, C**). This was in line with more CaOx crystal-induced tubular injury and interstitial fibrosis in WT B6N mice but not in the three knockout mouse strains following oxalate feeding, as illustrated by PAS (**Figure 1D**) and silver stain (**Supplementary Figure S1**). However, feeding an oxalate-rich diet did not lead to tubular injury, interstitial fibrosis and increased plasma BUN and creatinine levels in the three knockout mouse strains as compared to oxalate-fed B6N mice (**Figures 1B–D**, **Supplementary Figure S1**).

The adhesion of CaOx crystals on the surface of tubular epithelial cells is an important process in nephrocalcinosis (40). To look at potential changes of oxalate crystal-related adhesion molecules, we stained kidney sections with CD44 and Annexin II and found that the expression of CD44 and Annexin II increased in B6N mice on oxalate-rich diet after 14 days, whereas low expression of both adhesion molecules was observed in tubular cells in *Casp1* ^-/-^, *Cybb* ^-/-^ and *Casp1*/*Cybb* ^-/-^ mice (**Figures 1E, F**). Thus, the data indicate that unlike WT mice on a B6N background, *Casp1* ^-/-^, *Cybb* ^-/-^ and *Casp1*/*Cybb* ^-/-^ mice on a 129/B6J background lack CaOx crystal deposition and related nephrocalcinosis.

### B6N but Not B6J, 129 or Balb/c Mice Develop CaOx Crystal-Related Nephropathy

**Figure 2:**
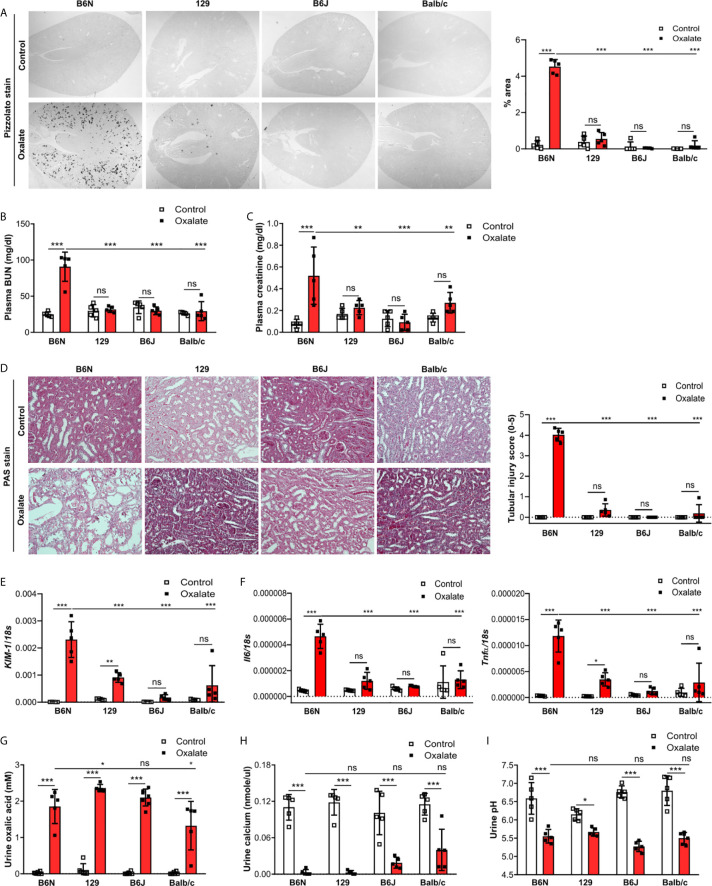
B6N but not B6J, 129 and Balb/c mice develop CaOx-related nephrocalcinosis. B6N, 129, B6J and Balb/c mice were either placed on a control diet or an oxalate-rich diet for 14 days. **(A)** Pizzolato staining to identify CaOx crystals and quantification (% area) of kidney sections. Original magnification ×25. **(B, C)** Plasma BUN **(B)** and plasma creatinine **(C)** levels were measured using colorimetric assays. **(D)** Images of PAS stained kidney sections and quantification of tubular injury score. Original magnification x200. **(E, F)** Gene expression of *KIM-1* **(E)** as well as *Il6 and Tnfα* **(F)** in kidney tissue determined by RT-PCR. **(G–I)** Urine was collected and the concentrations of oxalic acid **(G)** and calcium **(H)** as well as the urine pH **(I)** determined. Data are mean ± SD from 5 mice per group. *p < 0.05; **p < 0.01; ***p < 0.001; ns, not significant.

One possible reason for the absence of CaOx crystal in the *Casp1* ^-/-^, *Cybb* ^-/-^ and *Casp1*/*Cybb* ^-/-^ mice might be their genetic 129/B6J background rather than the specific gene deficiency. To investigate a potential contribution of the genetic background on CaOx crystal deposition and related nephrocalcinosis, four common inbred mouse strains B6N, B6J, 129 and Balb/c received an oxalate-rich diet for 14 days. Feeding B6N mice an oxalate-rich diet increased the percentage of Pizzolato area in kidney sections from B6N mice but not in 129, B6J and Balb/c mice (**Figure 2A**). While CaOx crystal deposition deteriorated the kidney function in B6N mice, as indicated by an increase in plasma BUN and creatinine levels (**Figures 2B, C**), no changes were observed in 129, B6J and Balb/c mice between both diets, indicating that those mice remained healthy due to the lack of CaOx crystal deposition. These findings are consistent with more tubular injury (**Figure 2D**), increased intrarenal mRNA expression levels of kidney injury marker (*KIM-1*) (**Figure 2E**) and inflammatory markers *Il6* and *Tnfα* (**Figure 2F**) as well as more interstitial fibrosis, as indicated by silver stain and mRNA expression levels of the fibrosis markers *Fibronectin 1* and *Col1a-1* (**Supplementary Figures S2A, B**) in B6N mice but not the other strains.

Next, we assessed urinary mineral concentrations of oxalic acid and calcium in the inbred mouse strains to rule out that differences in kidney oxalate excretion might be responsible for the observed discrepancy in CaOx crystal deposition. Urinary analysis revealed that feeding inbred mouse strains an oxalate-rich but calcium-free diet significantly increased the concentrations of oxalic acid (**Figure 2G**) but decreased that of urine calcium (**Figure 2H**) as well as urine pH (**Figure 2I**), suggesting that all four inbred mouse strains developed hyperoxaluria but only B6N mice presented with CaOx crystal deposits, an effect independent of the urine pH. Taken together, CaOx crystal-induced nephropathy in mice is sensitive to the genetic background with fundamental differences even between B6J and B6N.

### Increased Tubule Adhesion Molecule Expression in Oxalate-Fed B6N but Not B6J, 129 and Balb/c Mice

**Figure 3:**
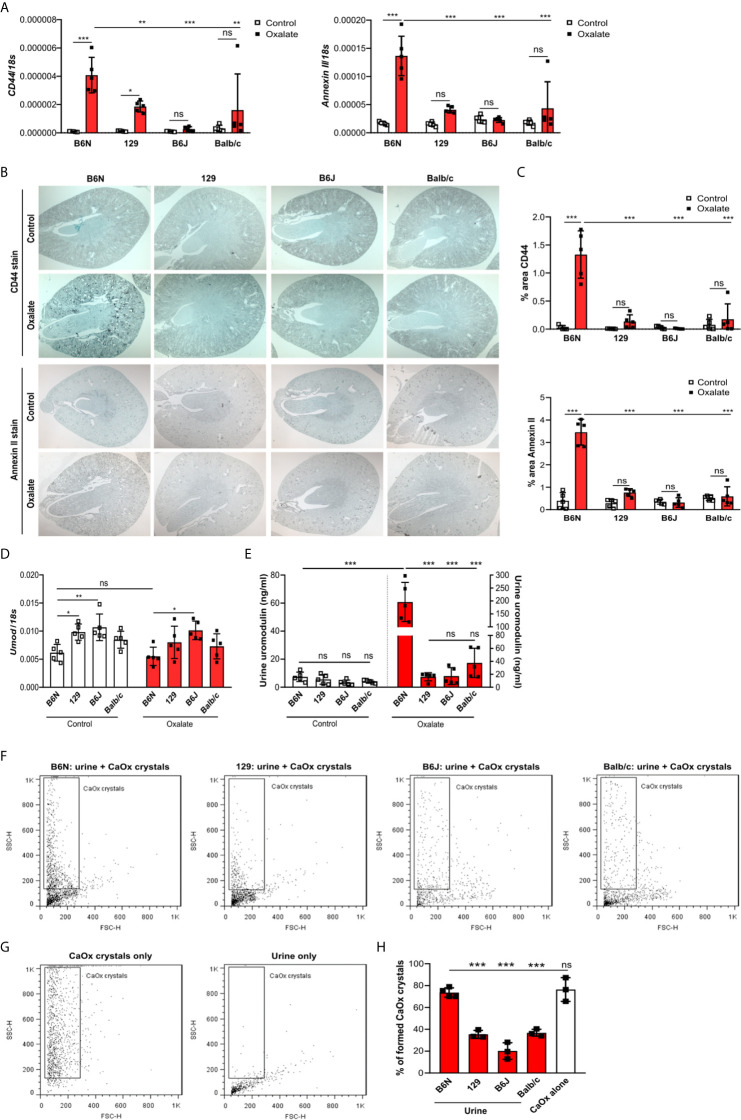
Increased urinary uromodulin excretion in B6N mice due to CaOx crystal deposition. **(A–E)** B6N, 129, B6J and Balb/c mice were either placed on a control diet or an oxalate-rich diet for 14 days. **(A)** Intrarenal mRNA expression of *CD44* and *Annexin II* determined by RT-PCR. **(B, C)** Immunostaining for CD44 and annexin II in kidney sections **(B)** and quantification of the percent (%) area **(C)**. Original magnification ×25. **(D)** Intrarenal mRNA expression of *Umod* in kidney tissues by RT-PCR. **(E)** Uromodulin concentration in urine measured by ELISA. Data are mean ± SD from 5 mice in each group. *p < 0.05; **p < 0.01; ***p < 0.001; ns, not significant. **(F–H)** CaOx crystallization assays *in vitro*. Urine from healthy B6N, 129, B6J and Balb/c mice was pre-incubated with or without 100 µl of a Na_2_C_2_O_4_ solution (oxalate, 0.1 mM, pH 7.3) for 1 hour at room temperature and then incubated with 100 µl CaCl_2_ buffer ﻿(0.1 mM, pH 7.3) for 5 minutes. Representative images of gating strategy to identify CaOx crystal formation in the presence or absence of mouse urine **(F, G)** and quantification of CaOx crystals by size (**H**, forward scatter vs. sideward scatter) using flow cytometry analysis. Data are mean ± SD from 3 mice per group. ***p < 0.001; ns indicates not significant.

To test whether tubule adhesion molecules (41, 42) that are required for the attachment of CaOx crystals to the tubular epithelial cell membrane, might be responsible for the inhibited CaOx crystal formation in B6J, 129 and Balb/c mice, we placed all four common inbred mouse strains on an oxalate-rich diet for 14 days and performed RT-PCR and immunohistochemistry staining of the kidneys. Intrarenal mRNA expression levels of ﻿*CD44* and *Annexin II* were lower in B6J, 129 and Balb/c mice as compared to B6N mice (**Figure 3A**). This was consistent with the immunostaining of kidney sections illustrating that CD44 and annexin II were highly expressed in B6N mice with CaOx crystal-induced nephropathy as compared to B6N mice on control diet (**Figures 3B, C**). However, oxalate feeding did not induce CD44 and annexin II expression in B6J, 129 and Balb/c mice (**Figures 3B, C**), suggesting that the different kidney oxalate handling and related CaOx crystal deposition depends on inbred mouse strains.

### Genetic Profiling Identifies Uromodulin as Determinant for CaOx Crystal-Induced Nephropathy

To investigate the genetic differences between the common inbred mouse strains, we first compared liver and kidney gene expression data between B6N versus 129 healthy mice as well as B6N versus B6J healthy mice. Open access liver microarray data (37) revealed that a total of 1211 genes (fold change cut-off ≥2 or ≤0.5) were differentially expressed in the liver between B6N and 129 mice, while only 5 genes between B6N and B6J mice (**Supplementary Figure S3A**). From 229 liver genes (fold change cut-off ≥3 or ≤0.3), we found 21 genes to be highly expressed in the mouse kidney and 26 genes with an intermediate expression according to the NCBI gene expression database, of which 20 and/or 24 genes were different in B6N mice as compared to 129 mice, while only 1 and/or 2 genes between B6N and B6J mice (**Supplementary Figure S3B**). Of the 21 highly expressed genes in the kidney, we selected 15 genes that are described in the literature to be associated with metabolism, transmembrane transport of small molecules and detoxification of reactive oxygen species pathways for further RT-PCR analysis. Intrarenal mRNA expression levels of certain enzymes such as sorbitol dehydrogenase (*Sord*), ornithine decarboxylase (*Odc1*) and heat shock protein 8 (*Hsp8*) were higher expressed in B6N and B6J mice as compared to 129 and Balb/c mice (**Supplementary Figure S3C**). The data demonstrate the genetic variations between the four common inbred mouse strains.

The pathophysiology underlying kidney stone formation is complex. Evidence points toward a genetic predisposition (43) for CaOx crystal formation and related nephrocalcinosis in human and mice (43–46). To test whether the observed differences in CaOx crystal deposition *in vivo* might be linked to kidney stone-related genes, we screened the literature for known nephrocalcinosis-related genes and performed RT-PCR of healthy kidneys from all four inbred mouse strains. As illustrated in the heat map in **Supplementary Figure S3D**, the mRNA expression levels of uromodulin (*Umod*) and sodium-dependent phosphate transport protein 2A (*Slc34a1*) were highly expressed, while others genes including organic cation transporter-2 (*Slc22a2*), 3-hydroxyisobutyrate dehydrogenase (*Hibadh*), sodium-chloride symporter (*Slc12a3*), aquaporin 1 (*Aqp1*), carbonic anhydrase II (*Car2*), ATP-binding cassette super-family G member (*Abcg2*) and urate transporter (*Slc22a12*) were lower expressed in healthy kidneys of all four inbred mouse strains. Looking at the mRNA expression levels in more detail, we found that NAD(P) transhydrogenase (*Nnt*), a previously described gene involved in nephrocalcinosis (5), to be absent in B6J mice but not in 129, B6N and Balb/c mice (**Supplementary Figure S4A**). In addition, healthy B6N mice had significantly lower *Umod* and oculocerebrorenal syndrome protein (*Ocrl*) mRNA expression levels in kidneys compared to the other three strains (**Figure 3D** and **Supplementary Figure S4B**).

Previous reports have shown that urinary proteins including uromodulin, also known as Tamm-Horsfall protein (THP), can affect CaOx crystal formation (47). We found that the intrarenal *Umod* mRNA expression levels were lower in B6N mice compared to the other strains upon feeding a control diet (**Figure 3D**), while no difference was observed in the urine concentration of uromodulin (**Figure 3E**). When feeding all strains an oxalate-rich diet, the intrarenal *Umod* mRNA expression levels did not change as compared to the control diet (**Figure 3D**). However, we observed a significant increase in the urine concentration of uromodulin in oxalate-fed B6N mice but not in the other strains as compared to control diet (**Figure 3E**). This was consistent with an increased THP/uromodulin positivity in kidney sections from B6N but not 129 mice after applying an oxalate-rich diet (**Supplementary Figure S4C**). We also noted that THP/uromodulin was highly expressed in healthy tubuli, while absent in areas where CaOx crystals (Pizzolato stain) were detected in kidney sections from oxalate-fed B6N mice (**Supplementary Figure S4D**).

To investigate the contribution of urinary constituents on CaOx crystal formation, we performed CaOx crystal formation experiments *in vitro* in the presence or absence of mouse urine and determined the percentage of formed CaOx crystals using flow cytometry (**Figures 3F, G**). Flow cytometry revealed that urine from B6N mice did not prevent CaOx crystal formation, as indicated by a similar percentage of CaOx crystals in the CaOx group without urine (**Figure 3H**). However, in the presence of urine from 129, B6J and Balb/c mice, CaOx crystals were significantly less able to form as compared to urine from B6N mice (**Figure 3H**), suggesting that urinary factors in 129, B6J and Balb/c mice prevents CaOx crystallization but not in B6N mice. Taken together, urinary factors can alter CaOx crystallization depending on the genetic background of mice and uromodulin excretion increases due to enhanced CaOx crystal deposition and renal clearance in B6N mice.

### Backcrossing of 129 Mice on a B6N Background Drives CaOx Crystal-Induced Nephropathy

**Figure 4:**
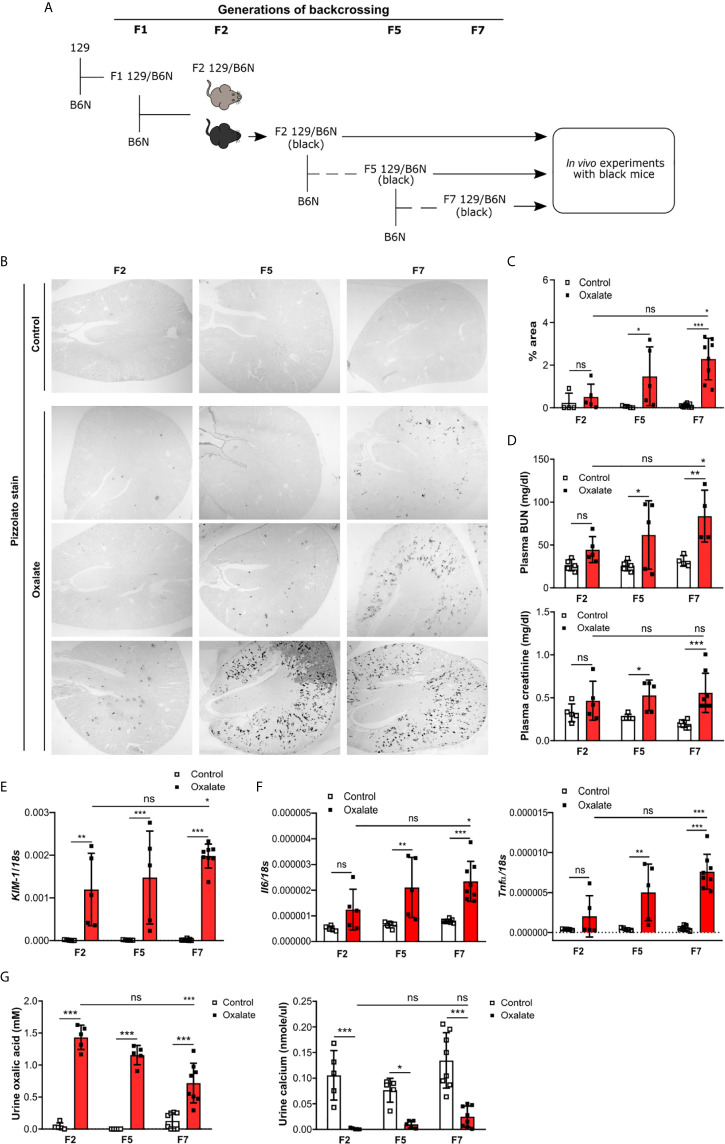
Backcrossing of 129 mice on a B6N background drives CaOx crystal-induced nephropathy. **(A)** Schematic diagram indicates the regime of generating 129/B6N mice by breeding 129 with B6N mice for up to 7 generations (F2, F5 and F7). Afterward, 129/B6N F2, F5 and F7 mice were either fed a control diet or an oxalate-rich diet for 14 days. **(B, C)** Pizzolato staining to identify CaOx crystal deposits in kidney sections **(B)** and the quantification **(C)**. Original magnification ×25. **(D)** Plasma BUN and creatinine levels were measured using colorimetric assays. **(E, F)** Intrarenal mRNA expression levels of *KIM-1* **(E)** as well as *Il6* and *Tnfα* **(F)** determined by RT-PCR. **(G)** Urinary concentrations of oxalic acid and calcium. Data are mean ± SD from 5 to 8 mice per group. *p < 0.05; **p < 0.01; ***p < 0.001; ns, not significant.

**Figure 5:**
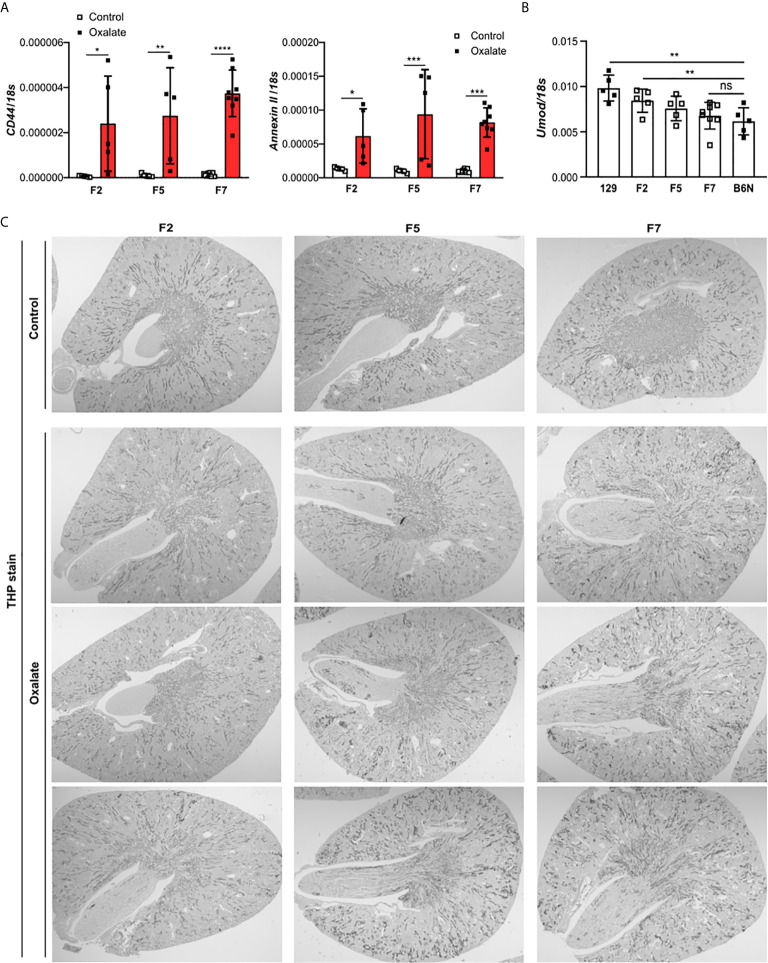
Uromodulin as indicator of the backcross generation in mice. 129/B6N mice were generated by breeding 129 with B6N mice for up to 7 generations (F2, F5 and F7). 129/B6N F2, F5 and F7 mice were either placed on a control diet or oxalate-rich diet for 14 days. **(A)** Intrarenal mRNA expression of *CD44* and *Annexin II* from mice on both diets determined by RT-PCR. **(B)** Intrarenal mRNA expression levels of *Umod* from mice on control diet by RT-PCR. **(C)** THP staining of kidney sections from all three generations of 129/B6N mice on day 14. Data are mean ± SD from 5 to 8 mice per group. *p < 0.05; **p < 0.01; ***p < 0.001; ****p < 0.0001. ns, not significant.

To dissect the interplay between the backcrossing of mice on a B6N background and the phenotypic development upon oxalate feeding, we generated 129/B6N mice by crossbreeding 129 mice with B6N mice for up to seven generations and placed them either on a control or oxalate-rich diet for 14 days (**Figure 4A**). We found that backcrossing 129 mice on a B6N background for two, five and seven generations (F2, F5 and F7) resulted occasionally in CaOx crystal deposition, although not in all 129/B6N mice from generation two and five, while all 129/B6N F7 mice showed CaOx crystal deposits similar to B6N mice (**Figures 4B, C**). CaOx crystal formation in 129/B6N mice was associated with a gradual increase in plasma BUN and creatinine levels (**Figure 4D**) due to tubular injury, as indicated by PAS stain (**Supplementary Figures S5A, B**) and intrarenal mRNA expression levels of *KIM-1* (**Figure 4E**), inflammation (**Figure 4F**) and interstitial fibrosis (**Supplementary Figures S5C, D**) after applying an oxalate-rich diet, although not significant from F2 to F5. All backcross generations of mice developed hyperoxaluria, as indicated by increased urinary oxalic acid and decreased urine calcium concentrations (**Figure 4G**). Consistent with these findings, we observed an upregulation of the mRNA expression levels of the adhesion molecules *CD44* and *Annexin II*, as a result of CaOx crystal deposition (**Figure 5A**). Further analysis showed that the intrarenal mRNA expression levels of *Umod* but not *Ocrl* gradually decreased in the healthy kidneys of 129/B6N F2, F5 and F7 mice, in which 129/B6N F7 mice reached similar levels to that observed in B6N mice (**Figure 5B** and **Supplemental Figure S6**). Immunohistochemistry staining of kidney sections illustrated increased THP expression in all 129/B6N mice from generation F7 as compared to the 2^nd^ (F2) and 5^th^ (F5) generations after applying an oxalate-rich diet (**Figure 5C**). Thus, backcrossing of 129 mice for a minimum of 7 generations on a B6N background leads to CaOx crystal deposition and subsequent CaOx crystal-induced nephropathy, suggesting that the genetic background is important for mice to develop phenotypic alterations.

### Co-Housing Does Not Influence CaOx Crystal Deposition in 129 Mice on a B6N Background

**Figure 6:**
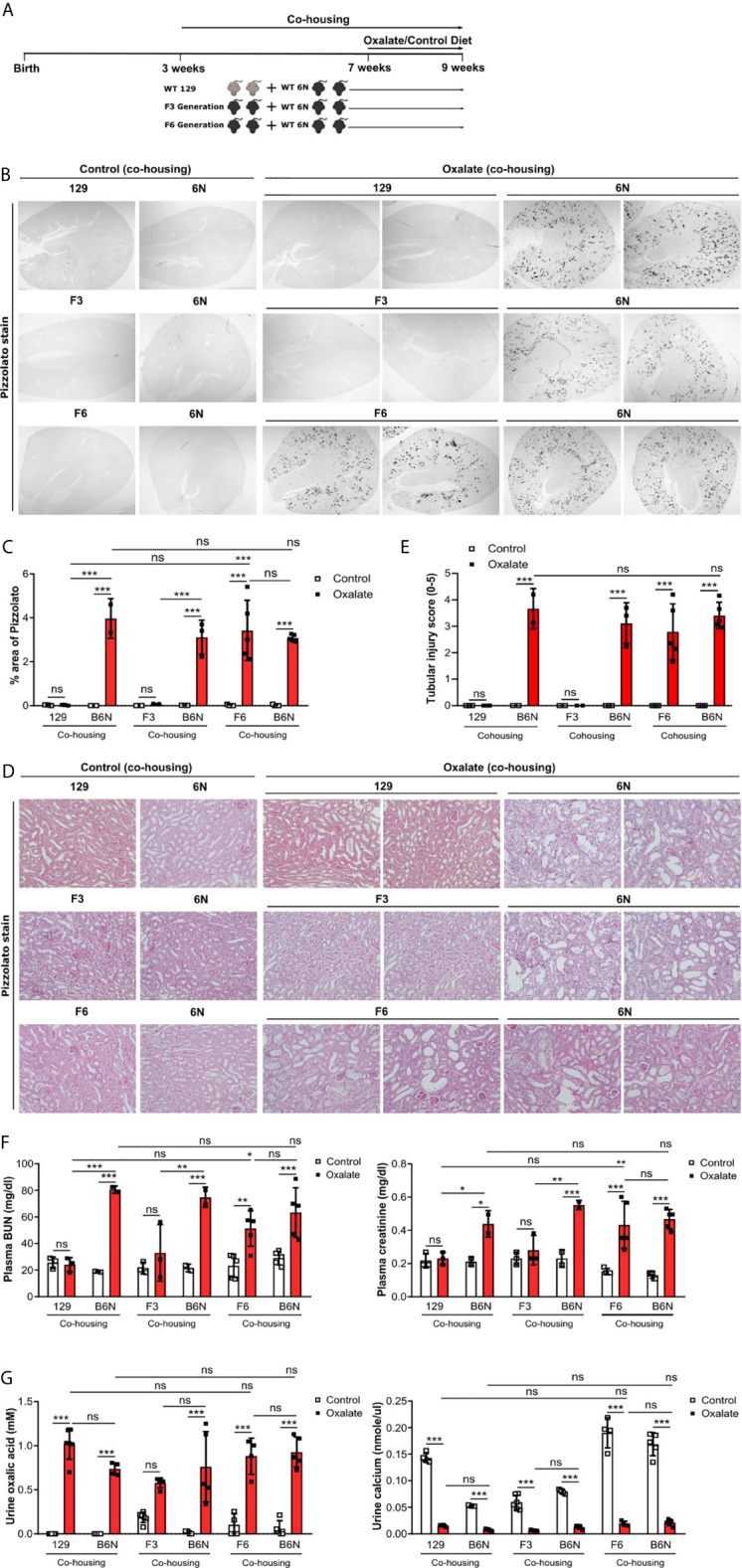
Co-housing does not influence CaOx crystal deposition in 129 mice on a B6N background. **(A)** Schematic diagram of experiment setup. Three week-old B6N mice were co-housed either with 129 mice or B6N/129 F3 and F6 mice for 4 weeks, and then placed on a control diet or oxalate-rich diet for 14 days. **(B, C)** Pizzolato staining to identify CaOx crystal deposits in kidney sections **(B)** and quantification of % area **(C)**. Original magnification ×25. **(D, E)** PAS stain illustrates tubular injury **(D)** with quantification **(E)**. Original magnification x200. **(F)** Plasma BUN and creatinine levels were measured by colorimetric assays. **(G)** Urinary concentrations of oxalic acid and calcium. Data are mean ± SD from 2 to 5 mice per group. *p < 0.05; **p < 0.01; ***p < 0.001; ns, not significant.

Previous studies reported a link between the intestinal microbiota with urinary oxalate excretion and CaOx crystal deposition in the context of hyperoxaluria (48). Therefore, we asked the question does the microbiome after co-housing contribute to CaOx crystal deposition in the kidneys? To answer this, 129 mice as well as 129/B6N F3 and F6 mice were co-housed with B6N mice for four weeks to allow adaptation of the microbiota (29) prior to oxalate feeding for 14 days (**Figure 6A**). We found that co-housing 129 and 129/B6N F3 mice with B6N mice did not trigger CaOx crystal deposition (**Figures 6B, C**). Only 129/B6N F6 mice co-housed with B6N mice presented with CaOx crystal deposits and subsequent nephrocalcinosis, as indicated by more tubular injury (**Figures 6D, E**) and interstitial fibrosis (**Supplementary Figure S7**) as well as increased plasma BUN and creatinine levels only in B6N mice but not in 129 mice after oxalate feeding (**Figure 6F**). On the other hand, backcrossed 129/B6N F6 mice showed similar tubular injury score, fibrosis and plasma BUN and creatinine levels as compared to B6N, whereas 129/B6N F3 mice did not (**Figures 6D–F**, **Supplementary Figure S7**). However, all mouse strains developed hyperoxaluria independent of co-housing and the genetic background, as indicated by increased urine oxalic acid and decreased urine calcium concentrations in all mice (**Figure 6G**). Taken together, only backcrossing to the optimal B6N background (genetics) but not co-housing (intestinal microbiome) determines CaOx crystal deposition and related nephrocalcinosis in mice.

## Discussion

We had hypothesized that the genetic background of mice and the intestinal microbiome after co-housing would affect CaOx crystal deposition in the kidneys and consequently the outcomes of CaOx-related nephrocalcinosis in four common inbred mouse strains. Indeed, only B6N mice but not 129, B6J and Balb/c mice developed nephrocalcinosis caused by the deposition of CaOx crystals. Backcrossing 129 mice with B6N for up to seven generations restored CaOx crystal deposition and induced nephrocalcinosis, a process associated with uromodulin levels. Thus, genetic variations across mouse strains independent of co-housing may account for phenotypic differences observed in mouse models of CaOx crystal-induced nephropathy.

Targeted mutation in mice is a powerful tool for functional analysis of genes. However, genetic variations in mice that are linked to the origin of ES cells (mostly derived from 129 mice), the number of backcrosses (e.g. 7-10 generations) of genetically modified mice with inbred mouse strains such as the ﻿C57BL/6 substrains B6N or B6J, as well as the breeding strategies across generations might influence the phenotypic outcomes (49). For example, B6J mice are more susceptible to lipopolysaccharide- and tumor necrosis factor α-induced lethal shock as compared to B6N/B6J mice (16). In tumor development, the lymphoma formation in *Pten*-deficient mice depends on C57BL/6 background, but not Balb/c background (50). When studying the role of JNK2 in Acetaminophen and Concanavalin A liver injury, mispairing C57BL/6 strains of genetically engineered mice and wild-type controls can lead to confounding results (51). In a mouse model of nephrocalcinosis, we observed that only B6N but not *Casp1*-, *Cybb*- and *Casp1/Cybb*-deficient mice developed CaOx crystal-induced nephropathy. One explanation for the lack of CaOx crystal deposition is that these genetically modified mice were generated in 129- or D3-derived ES cells (52, 53) and not backcrossed for more than 7 generations on a B6N background. Therefore, it is impossible to conclude on a role of caspase-1 and Cybb in CaOx crystal-induced nephropathy. Possible technical alternatives to overcome this issue could be to use multiplex genome engineering technologies such as CRISPR/Cas systems (54–56) or cre ES cell lines derived from B6N or B6J mice (16), which will enable researchers to use genetically modified mice with a defined genetic background and thus, to circumvent problems associated with phenotypic interference and misinterpretation of functional data.

The mechanism of CaOx crystallization involves a combination of processes, including urine supersaturation of stone-forming salts, such as calcium and oxalate (35, 39, 57). Our data imply that low calcium and high oxalate levels are indicators for the development of hyperoxaluria in all four inbred mouse strains after feeding an oxalate-rich diet. Although, oxalate feeding induced an acidic urine pH in all four inbred mouse strains, only B6N showed CaOx crystal deposits in the kidney but not 129, B6J and Balb/c mice, suggesting that the urine pH may not be involved in CaOx crystal formation. This is consistent with reports indicating that CaOx supersaturation occurs independent of urine pH (3), while in other types of kidney stone disease, an acidic urine pH seems to be necessary for the formation of e.g. uric acid crystals, which in turn causes chronic uric acid crystal nephropathy (9).

Genetic variations play a critical role in CaOx crystal formation. For example, Masayuki et al. reported in a model of acute CaOx nephropathy that B6J mice have more kidney CaOx crystal deposition than B6N mice when administered with glyoxylate daily for 12 days (5). The authors suggest that the absence of the Nnt protein is associated with CaOx crystal formation in B6J mice. However, in our study, B6J mice lacking *Nnt* expression failed to develop chronic CaOx crystal nephropathy, while B6N mice with high *Nnt* expression did. On the other hand, 129 and Balb/c mice with high *Nnt* gene expression similar to B6N mice mostly failed to develop CaOx crystal formation, suggesting that Nnt might not be involved in CaOx crystal formation and that the background of genetically modified mice plays a critical role on the functional phenotype in the disease setting. Although we screened for known genes associated with nephrocalcinosis in mice and humans (43–46), functional studies are needed to identify specific genes that either prevent or promote CaOx crystallization *in vivo*.

Uromodulin is the most abundant urinary protein in healthy humans. This extracellular matrix-type protein is produced and excreted by epithelial cells lining the thick ascending limb of the loop of Henle (58). Important roles for uromodulin include protection against urinary tract infections by binding to type 1-fimbriated uropathogenic *E. coli* (59, 60) and reduction of kidney stone formation by binding CaOx crystals (47, 61). We found significantly elevated urinary protein levels of uromodulin in B6N mice compared with the other strains upon oxalate feeding, while the kidney mRNA expression levels remained unchanged. CaOx-related nephropathy in the face of increased urine uromodulin appears paradox. However, similar results were reported in a mouse model of ﻿unilateral ureteral obstruction-induced kidney injury without crystal formation, where kidney and urinary uromodulin protein levels increased but not kidney *Umod* mRNA levels (62). This might be due to intrarenal uromodulin protein retention upon kidney injury because 1) uromodulin is continuously produced and excreted by the kidney, and can form disulfide bond-rich tertiary structures with the tendency to polymerize, and 2) the remaining unaffected tubular epithelial cells produce large amounts of uromodulin, while injured tubular epithelial cells cannot anymore, leading to increased urinary uromodulin excretion. Further studies are needed to verify this in uromodulin knockout mice and humans.

The mammalian intestines harbor a complex and variable microbial ecosystem, which is strongly associated with the health status and well-being. Prior investigations in animals as well as clinical data have demonstrated that certain components of the intestinal microbiota are a strong determinant of kidney stone disease (48, 63–66). For example, certain bacterial strains, including *Oxalobacter* spp., *Bifidobacterium* spp. and *Lactobacillus* spp., can degrade oxalate (66, 67). Moreover, intestinal bacteria might not only alter oxalate metabolism but also increase oxalate secretion from intestinal epithelia (68, 69). Numerous studies have reported various bacterial strains and specific variations in bacteria taxa among different inbred mouse lines (70–73). Co-housing of different mouse strains can partially shape the intestinal bacterial consortia in the fecal pellet (29), in which bacterial communities became more similar but retained strain specificity (72). Our findings show that co-housing has no effect on CaOx crystal deposition ﻿in a mouse model of nephrocalcinosis, a process potentially linked to the genetic background of mice. To minimize or address the influence of the microbiota in preclinical genotype-phenotype studies, co-housing animals followed by inter-crossing to generate F2 littermates could be used as a standard tool to assure adaptation of the microbiome of the animals of different treatment groups (29). However, we cannot exclude that co-housing of backcrossed inbred mice with B6N mice may have shifted the microbiota composition.

In summary, our findings suggest that all inbred mouse strains develop hyperoxaluria but exhibit differences in both CaOx crystallization and kidney injury following administration of an oxalate-rich diet. Genetic variations among mouse strains might be responsible in part for strain-specific CaOx crystal formation and that urinary factors such as uromodulin can act as a determinant of CaOx-related nephropathy in common inbred mouse strains. Thus, researchers using knockout mice of differing genetic backgrounds should be aware that any variation in CaOx crystal formation and associated kidney immune response might lead to misinterpretations of data among studies. In particular, phenotype differences between knockout and WT mice can only be attributed to the targeted gene when using littermates of the identical genetic background. In contrast, co- or separate housing does not seem to be a relevant confounder in mice.

## Data Availability Statement

The raw data supporting the conclusions of this article will be made available by the authors, without undue reservation.

## Ethics Statement

The animal study was reviewed and approved by Regierung von Oberbayern. All animal experiments were approved by the local government authorities Regierung von Oberbayern 416 (reference number: ROB-55.2-2532.Vet_02-15-189 and 02-18-127) and Berlin in accordance with the 417 European directive for the Protection of Animals Used for Scientific Purposes (2010/63/EU) and 418 reported according to the ARRIVE guidelines (74).

## Author Contributions

QM and SS designed the study. QM, MG, and SS conducted experiments. ML performed the open access gene expression profiles data analysis. AS and RK provided the transgenic mice. QM and SS analyzed and interpreted the data. QM, H-JA and SS wrote the manuscript. All authors contributed to the article and approved the submitted version.

## Funding

The work was supported by grants from the Deutsche Forschungsgemeinschaft to SS (STE2437/2-1 and 2-2) and to H-JA (AN372/16-2, 24-1, 27-1).

## Conflict of Interest

The authors declare that the research was conducted in the absence of any commercial or financial relationships that could be construed as a potential conflict of interest.

## References

[1] Coe FL Evan A Worcester E. Kidney Stone Disease. J Clin Invest (2005) 115(10):2598–608. 10.1172/JCI26662 PMC1236703 16200192

[2] Mulay SR Anders HJ. Crystallopathies. N Engl J Med (2016) 375(13):e29. 10.1056/NEJMc1609332 27682056

[3] Carvalho M. Urinary Ph in Calcium Oxalate Stone Formers: Does It Matter? J Bras Nefrol (2018) 40(1):6–7. 10.1590/1678-4685-jbn-2018-00010002 29738024 PMC6533969

[4] Khan SR Pearle MS Robertson WG Gambaro G Canales BK Doizi S. Kidney Stones. Nat Rev Dis Primers (2016) 2:16008. 10.1038/nrdp.2016.8 27188687 PMC5685519

[5] Usami M Okada A Taguchi K Hamamoto S Kohri K Yasui T. Genetic Differences in C57BL/6 Mouse Substrains Affect Kidney Crystal Deposition. Urolithiasis (2018) 46(6):515–22. 10.1007/s00240-018-1040-3 29362828

[6] Mulay SR Anders HJ. Crystal Nephropathies: Mechanisms of Crystal-Induced Kidney Injury. Nat Rev Nephrol (2017) 13(4):226–40. 10.1038/nrneph.2017.10 28218266

[7] Goldfarb DS Fischer ME Keich Y Goldberg J. A Twin Study of Genetic and Dietary Influences on Nephrolithiasis: A Report From the Vietnam Era Twin (VET) Registry. Kidney Int (2005) 67(3):1053–61. 10.1111/j.1523-1755.2005.00170.x 15698445

[8] Curhan GC Willett WC Rimm EB Stampfer MJ. Family History and Risk of Kidney Stones. J Am Soc Nephrol (1997) 8(10):1568–73. 10.1681/ASN.V8101568 9335385

[9] Sellmayr M Hernandez Petzsche MR Ma Q Kruger N Liapis H Brink A. Only Hyperuricemia With Crystalluria, But Not Asymptomatic Hyperuricemia, Drives Progression of Chronic Kidney Disease. J Am Soc Nephrol (2020) 31(12):2773–92. 10.1681/ASN.2020040523 PMC7790211 32938648

[10] Klinkhammer BM Djudjaj S Kunter U Palsson R Edvardsson VO Wiech T. Cellular and Molecular Mechanisms of Kidney Injury in 2,8-Dihydroxyadenine Nephropathy. J Am Soc Nephrol (2020) 31(4):799–816. 10.1681/ASN.2019080827 32086278 PMC7191925

[11] Breiderhoff T Himmerkus N Stuiver M Mutig K Will C Meij IC. Deletion of claudin-10 (Cldn10) in the Thick Ascending Limb Impairs Paracellular Sodium Permeability and Leads to Hypermagnesemia and Nephrocalcinosis. Proc Natl Acad Sci USA (2012) 109(35):14241–6. 10.1073/pnas.1203834109 PMC3435183 22891322

[12] Jiang Z Asplin JR Evan AP Rajendran VM Velazquez H Nottoli TP. Calcium Oxalate Urolithiasis in Mice Lacking Anion Transporter Slc26a6. Nat Genet (2006) 38(4):474–8. 10.1038/ng1762 16532010

[13] Sun L Zou LX Wang J Chen T Han YC Zhu DD. Mucin 4 Gene Silencing Reduces Oxidative Stress and Calcium Oxalate Crystal Formation in Renal Tubular Epithelial Cells Through the Extracellular Signal-Regulated Kinase Signaling Pathway in Nephrolithiasis Rat Model. Kidney Blood Press Res (2018) 43(3):820–35. 10.1159/000490136 29843125

[14] Mekada K Yoshiki A. Substrains Matter in Phenotyping of C57BL/6 Mice. Exp Anim (2021). 10.1538/expanim.20-0158 PMC8150240 33441510

[15] Nagy A Vintersten K. Murine Embryonic Stem Cells. Methods Enzymol (2006) 418:3–21. 10.1016/S0076-6879(06)18001-5 17141026

[16] Vanden Berghe T Hulpiau P Martens L Vandenbroucke RE Van Wonterghem E Perry SW. Passenger Mutations Confound Interpretation of All Genetically Modified Congenic Mice. Immunity (2015) 43(1):200–9. 10.1016/j.immuni.2015.06.011 PMC4800811 26163370

[17] Rivera J Tessarollo L. Genetic Background and the Dilemma of Translating Mouse Studies to Humans. Immunity (2008) 28(1):1–4. 10.1016/j.immuni.2007.12.008 18199409

[18] Lim JP Zou ME Janak PH Messing RO. Responses to Ethanol in C57BL/6 Versus C57BL/6 X 129 Hybrid Mice. Brain Behav (2012) 2(1):22–31. 10.1002/brb3.29 22574271 PMC3343296

[19] Dobrowolski P Fischer M Naumann R. Novel Insights Into the Genetic Background of Genetically Modified Mice. Transgenic Res (2018) 27(3):265–75. 10.1007/s11248-018-0073-2 29663254

[20] Denburg MR Koepsell K Lee JJ Gerber J Bittinger K Tasian GE. Perturbations of the Gut Microbiome and Metabolome in Children With Calcium Oxalate Kidney Stone Disease. J Am Soc Nephrol (2020) 31(6):1358–69. 10.1681/ASN.2019101131 PMC7269359 32381601

[21] Stern JM Moazami S Qiu Y Kurland I Chen Z Agalliu I. Evidence for a Distinct Gut Microbiome in Kidney Stone Formers Compared to Non-Stone Formers. Urolithiasis (2016) 44(5):399–407. 10.1007/s00240-016-0882-9 27115405 PMC8887828

[22] Tang R Jiang Y Tan A Ye J Xian X Xie Y. 16S RRNA Gene Sequencing Reveals Altered Composition of Gut Microbiota in Individuals With Kidney Stones. Urolithiasis (2018) 46(6):503–14. 10.1007/s00240-018-1037-y 29353409

[23] Ticinesi A Milani C Guerra A Allegri F Lauretani F Nouvenne A. Understanding the Gut-Kidney Axis in Nephrolithiasis: An Analysis of the Gut Microbiota Composition and Functionality of Stone Formers. Gut (2018) 67(12):2097–106. 10.1136/gutjnl-2017-315734 29705728

[24] Ticinesi A Nouvenne A Meschi T. Gut Microbiome and Kidney Stone Disease: Not Just an Oxalobacter Story. Kidney Int (2019) 96(1):25–7. 10.1016/j.kint.2019.03.020 31229040

[25] Knauf F Brewer JR Flavell RA. Immunity, Microbiota and Kidney Disease. Nat Rev Nephrol (2019) 15(5):263–74. 10.1038/s41581-019-0118-7 30796361

[26] Ferraro PM Curhan GC Gambaro G Taylor EN. Antibiotic Use and Risk of Incident Kidney Stones in Female Nurses. Am J Kidney Dis (2019) 74(6):736–41. 10.1053/j.ajkd.2019.06.005 PMC7006481 31543288

[27] Tasian GE Jemielita T Goldfarb DS Copelovitch L Gerber JS Wu Q. Oral Antibiotic Exposure and Kidney Stone Disease. J Am Soc Nephrol (2018) 29(6):1731–40. 10.1681/ASN.2017111213 PMC6054354 29748329

[28] Schreiber A Luft FC Kettritz R. Phagocyte NADPH Oxidase Restrains the Inflammasome in ANCA-induced Gn. J Am Soc Nephrol (2015) 26(2):411–24. 10.1681/ASN.2013111177 PMC4310655 25012177

[29] Robertson SJ Lemire P Maughan H Goethel A Turpin W Bedrani L. Comparison of Co-Housing and Littermate Methods for Microbiota Standardization in Mouse Models. Cell Rep (2019) 27(6):1910–9.e2. 10.1016/j.celrep.2019.04.023 31067473

[30] McCoy KD Geuking MB Ronchi F. Gut Microbiome Standardization in Control and Experimental Mice. Curr Protoc Immunol (2017) 117:23 1 1–23 1 13. 10.1002/cpim.25 28369684

[31] Ma Q Steiger S Anders HJ. Sodium Glucose Transporter-2 Inhibition Has No Renoprotective Effects on Non-Diabetic Chronic Kidney Disease. Physiol Rep (2017) 5(7):e13228. 10.14814/phy2.13228 28364032 PMC5392518

[32] Mulay SR Eberhard JN Pfann V Marschner JA Darisipudi MN Daniel C. Oxalate-Induced Chronic Kidney Disease With Its Uremic and Cardiovascular Complications in C57BL/6 Mice. Am J Physiol Renal Physiol (2016) 310(8):F785–95. 10.1152/ajprenal.00488.2015 PMC5504458 26764204

[33] Anders HJ Suarez-Alvarez B Grigorescu M Foresto-Neto O Steiger S Desai J. The Macrophage Phenotype and Inflammasome Component NLRP3 Contributes to Nephrocalcinosis-Related Chronic Kidney Disease Independent From IL-1-mediated Tissue Injury. Kidney Int (2018) 93(3):656–69. 10.1016/j.kint.2017.09.022 29241624

[34] Marschner JA Mulay SR Steiger S Anguiano L Zhao Z Boor P. The Long Pentraxin PTX3 is an Endogenous Inhibitor of Hyperoxaluria-Related Nephrocalcinosis and Chronic Kidney Disease. Front Immunol (2018) 9:2173. 10.3389/fimmu.2018.02173 30319631 PMC6167460

[35] Steiger S Grill JF Ma Q Bauerle T Jordan J Smolle M. Anti-Transforming Growth Factor Beta Igg Elicits a Dual Effect on Calcium Oxalate Crystallization and Progressive Nephrocalcinosis-Related Chronic Kidney Disease. Front Immunol (2018) 9:619. 10.3389/fimmu.2018.00619 29651290 PMC5884871

[36] Mulay SR Kulkarni OP Rupanagudi KV Migliorini A Darisipudi MN Vilaysane A. Calcium Oxalate Crystals Induce Renal Inflammation by NLRP3-mediated IL-1beta Secretion. J Clin Invest (2013) 123(1):236–46. 10.1172/JCI63679 PMC3533282 23221343

[37] Kahle M Horsch M Fridrich B Seelig A Schultheiss J Leonhardt J. Phenotypic Comparison of Common Mouse Strains Developing High-Fat Diet-Induced Hepatosteatosis. Mol Metab (2013) 2(4):435–46. 10.1016/j.molmet.2013.07.009 PMC3855089 24327959

[38] Tusher VG Tibshirani R Chu G. Significance Analysis of Microarrays Applied to the Ionizing Radiation Response. Proc Natl Acad Sci USA (2001) 98(9):5116–21. 10.1073/pnas.091062498 PMC33173 11309499

[39] Thongboonkerd V Semangoen T Chutipongtanate S. Factors Determining Types and Morphologies of Calcium Oxalate Crystals: Molar Concentrations, Buffering, pH, Stirring and Temperature. Clin Chim Acta (2006) 367(1-2):120–31. 10.1016/j.cca.2005.11.033 16458875

[40] Taguchi K Okada A Kitamura H Yasui T Naiki T Hamamoto S. Colony-Stimulating Factor-1 Signaling Suppresses Renal Crystal Formation. J Am Soc Nephrol (2014) 25(8):1680–97. 10.1681/ASN.2013060675 PMC4116057 24578130

[41] Kumar V Farell G Deganello S Lieske JC. Annexin II is Present on Renal Epithelial Cells and Binds Calcium Oxalate Monohydrate Crystals. J Am Soc Nephrol (2003) 14(2):289–97. 10.1097/01.ASN.0000046030.24938.0A 12538728

[42] Verkoelen CF Verhulst A. Proposed Mechanisms in Renal Tubular Crystal Retention. Kidney Int (2007) 72(1):13–8. 10.1038/sj.ki.5002272 17429341

[43] Sayer JA. Progress in Understanding the Genetics of Calcium-Containing Nephrolithiasis. J Am Soc Nephrol (2017) 28(3):748–59. 10.1681/ASN.2016050576 PMC5328168 27932479

[44] Vezzoli G Terranegra A Arcidiacono T Soldati L. Genetics and Calcium Nephrolithiasis. Kidney Int (2011) 80(6):587–93. 10.1038/ki.2010.430 20962745

[45] Howles SA Wiberg A Goldsworthy M Bayliss AL Gluck AK Ng M. Genetic Variants of Calcium and Vitamin D Metabolism in Kidney Stone Disease. Nat Commun (2019) 10(1):5175. 10.1038/s41467-019-13145-x 31729369 PMC6858460

[46] Okada A Yasui T Hamamoto S Hirose M Kubota Y Itoh Y. Genome-Wide Analysis of Genes Related to Kidney Stone Formation and Elimination in the Calcium Oxalate Nephrolithiasis Model Mouse: Detection of Stone-Preventive Factors and Involvement of Macrophage Activity. J Bone Miner Res (2009) 24(5):908–24. 10.1359/jbmr.081245 19113933

[47] Mo L Huang HY Zhu XH Shapiro E Hasty DL Wu XR. Tamm-Horsfall Protein is a Critical Renal Defense Factor Protecting Against Calcium Oxalate Crystal Formation. Kidney Int (2004) 66(3):1159–66. 10.1111/j.1523-1755.2004.00867.x 15327412

[48] Hatch M. Gut Microbiota and Oxalate Homeostasis. Ann Transl Med (2017) 5(2):36. 10.21037/atm.2016.12.70 28217701 PMC5300851

[49] Farkas C Fuentes-Villalobos F Rebolledo-Jaramillo B Benavides F Castro AF Pincheira R. Streamlined Computational Pipeline for Genetic Background Characterization of Genetically Engineered Mice Based on Next Generation Sequencing Data. BMC Genomics (2019) 20(1):131. 10.1186/s12864-019-5504-9 30755158 PMC6373082

[50] Freeman D Lesche R Kertesz N Wang S Li G Gao J. Genetic Background Controls Tumor Development in PTEN-deficient Mice. Cancer Res (2006) 66(13):6492–6. 10.1158/0008-5472.CAN-05-4143 16818619

[51] Bourdi M Davies JS Pohl LR. Mispairing C57BL/6 Substrains of Genetically Engineered Mice and Wild-Type Controls Can Lead to Confounding Results as It Did in Studies of JNK2 in Acetaminophen and Concanavalin a Liver Injury. Chem Res Toxicol (2011) 24(6):794–6. 10.1021/tx200143x PMC3157912 21557537

[52] Kuida K Lippke JA Ku G Harding MW Livingston DJ Su MS. Altered Cytokine Export and Apoptosis in Mice Deficient in Interleukin-1 Beta Converting Enzyme. Science (1995) 267(5206):2000–3. 10.1126/science.7535475 7535475

[53] Pollock JD Williams DA Gifford MA Li LL Du X Fisherman J. Mouse Model of X-Linked Chronic Granulomatous Disease, an Inherited Defect in Phagocyte Superoxide Production. Nat Genet (1995) 9(2):202–9. 10.1038/ng0295-202 7719350

[54] Cong L Ran FA Cox D Lin S Barretto R Habib N. Multiplex Genome Engineering Using CRISPR/Cas Systems. Science (2013) 339(6121):819–23. 10.1126/science.1231143 PMC3795411 23287718

[55] Jinek M Chylinski K Fonfara I Hauer M Doudna JA Charpentier E. A Programmable Dual-RNA-guided DNA Endonuclease in Adaptive Bacterial Immunity. Science (2012) 337(6096):816–21. 10.1126/science.1225829 PMC6286148 22745249

[56] Platt RJ Chen S Zhou Y Yim MJ Swiech L Kempton HR. CRISPR-Cas9 Knockin Mice for Genome Editing and Cancer Modeling. Cell (2014) 159(2):440–55. 10.1016/j.cell.2014.09.014 PMC4265475 25263330

[57] Manissorn J Fong-Ngern K Peerapen P Thongboonkerd V. Systematic Evaluation for Effects of Urine Ph on Calcium Oxalate Crystallization, Crystal-Cell Adhesion and Internalization Into Renal Tubular Cells. Sci Rep (2017) 7(1):1798. 10.1038/s41598-017-01953-4 28496123 PMC5431959

[58] Devuyst O Olinger E Rampoldi L. Uromodulin: From Physiology to Rare and Complex Kidney Disorders. Nat Rev Nephrol (2017) 13(9):525–44. 10.1038/nrneph.2017.101 28781372

[59] Mo L Zhu XH Huang HY Shapiro E Hasty DL Wu XR. Ablation of the Tamm-Horsfall Protein Gene Increases Susceptibility of Mice to Bladder Colonization by Type 1-Fimbriated Escherichia Coli. Am J Physiol Renal Physiol (2004) 286(4):F795–802. 10.1152/ajprenal.00357.2003 14665435

[60] Bates JM Raffi HM Prasadan K Mascarenhas R Laszik Z Maeda N. Tamm-Horsfall Protein Knockout Mice are More Prone to Urinary Tract Infection: Rapid Communication. Kidney Int (2004) 65(3):791–7. 10.1111/j.1523-1755.2004.00452.x 14871399

[61] Liu Y Mo L Goldfarb DS Evan AP Liang F Khan SR. Progressive Renal Papillary Calcification and Ureteral Stone Formation in Mice Deficient for Tamm-Horsfall Protein. Am J Physiol Renal Physiol (2010) 299(3):F469–78. 10.1152/ajprenal.00243.2010 PMC2944300 20591941

[62] Maydan O McDade PG Liu Y Wu XR Matsell DG Eddy AA. Uromodulin Deficiency Alters Tubular Injury and Interstitial Inflammation But Not Fibrosis in Experimental Obstructive Nephropathy. Physiol Rep (2018) 6(6):e13654. 10.14814/phy2.13654 29595914 PMC5875544

[63] Al-Wahsh I Wu Y Liebman M. Acute Probiotic Ingestion Reduces Gastrointestinal Oxalate Absorption in Healthy Subjects. Urol Res (2012) 40(3):191–6. 10.1007/s00240-011-0421-7 21874572

[64] Knight J Deora R Assimos DG Holmes RP. The Genetic Composition of Oxalobacter Formigenes and Its Relationship to Colonization and Calcium Oxalate Stone Disease. Urolithiasis (2013) 41(3):187–96. 10.1007/s00240-013-0566-7 PMC3713771 23632911

[65] Mehta M Goldfarb DS Nazzal L. The Role of the Microbiome in Kidney Stone Formation. Int J Surg (2016) 36(Pt D):607–12. 10.1016/j.ijsu.2016.11.024 PMC5764756 27847292

[66] Turroni S Bendazzoli C Dipalo SC Candela M Vitali B Gotti R. Oxalate-Degrading Activity in Bifidobacterium Animalis Subsp. Lactis: Impact of Acidic Conditions on the Transcriptional Levels of the Oxalyl Coenzyme a (CoA) Decarboxylase and formyl-CoA Transferase Genes. Appl Environ Microbiol (2010) 76(16):5609–20. 10.1128/AEM.00844-10 PMC2918965 20601517

[67] Klimesova K Whittamore JM Hatch M. Bifidobacterium Animalis Subsp. Lactis Decreases Urinary Oxalate Excretion in a Mouse Model of Primary Hyperoxaluria. Urolithiasis (2015) 43(2):107–17. 10.1007/s00240-014-0728-2 PMC4629830 25269440

[68] Hatch M Cornelius J Allison M Sidhu H Peck A Freel RW. Oxalobacter Sp. Reduces Urinary Oxalate Excretion by Promoting Enteric Oxalate Secretion. Kidney Int (2006) 69(4):691–8. 10.1038/sj.ki.5000162 16518326

[69] Hatch M Freel RW Vaziri ND. Regulatory Aspects of Oxalate Secretion in Enteric Oxalate Elimination. J Am Soc Nephrol (1999) 10(Suppl 14):S324–8. 10541256

[70] Deloris Alexander A Orcutt RP Henry JC Baker J, Jr. Bissahoyo AC Threadgill DW. Quantitative PCR Assays for Mouse Enteric Flora Reveal Strain-Dependent Differences in Composition That are Influenced by the Microenvironment. Mamm Genome (2006) 17(11):1093–104. 10.1007/s00335-006-0063-1 17091319

[71] Friswell MK Gika H Stratford IJ Theodoridis G Telfer B Wilson ID. Site and Strain-Specific Variation in Gut Microbiota Profiles and Metabolism in Experimental Mice. PloS One (2010) 5(1):e8584. 10.1371/journal.pone.0008584 20052418 PMC2798964

[72] Campbell JH Foster CM Vishnivetskaya T Campbell AG Yang ZK Wymore A. Host Genetic and Environmental Effects on Mouse Intestinal Microbiota. ISME J (2012) 6(11):2033–44. 10.1038/ismej.2012.54 PMC3475380 22695862

[73] Hildebrand F Nguyen TL Brinkman B Yunta RG Cauwe B Vandenabeele P. Inflammation-Associated Enterotypes, Host Genotype, Cage and Inter-Individual Effects Drive Gut Microbiota Variation in Common Laboratory Mice. Genome Biol (2013) 14(1):R4. 10.1186/gb-2013-14-1-r4 23347395 PMC4053703

[74] Kilkenny C Browne WJ Cuthill IC Emerson M Altman DG. Improving Bioscience Research Reporting: The ARRIVE Guidelines for Reporting Animal Research. PloS Biol (2010) 8(6):e1000412. 10.1371/journal.pbio.1000412 20613859 PMC2893951

