# Supplementary material for: Genetic Background but Not Intestinal Microbiota After Co-Housing Determines Hyperoxaluria-Related Nephrocalcinosis in Common Inbred Mouse Strains
Source: Front Immunol. 2021 Apr 21;12:673423. doi: 10.3389/fimmu.2021.673423 (PMC8100042; doi:10.3389/fimmu.2021.673423)
Supplement: Supplementary file 1 [file DataSheet_1.pdf]

Supplementary Material

1 Supplementary Figures and Tables

1.1 Supplementary Figures

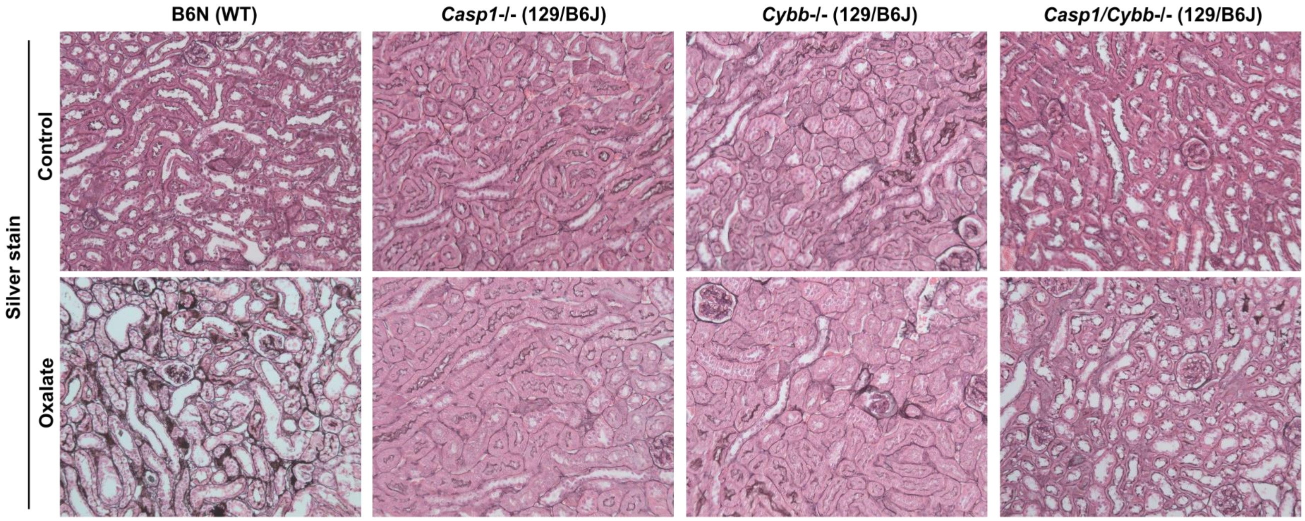

Supplementary Figure S1. Knockout mice on a 129/B6J background do not display interstitial fibrosis upon feeding an oxalate-rich diet. C57BL/6N WT (B6N) and *Casp1*-, *Cybb*-, *Casp1/Cybb*-deficient (129/B6J background) mice were either fed a control diet or an oxalate-rich diet for 14 days. Silver staining to illustrate interstitial fibrosis in kidney sections of all four mouse strains. Original magnification x200.

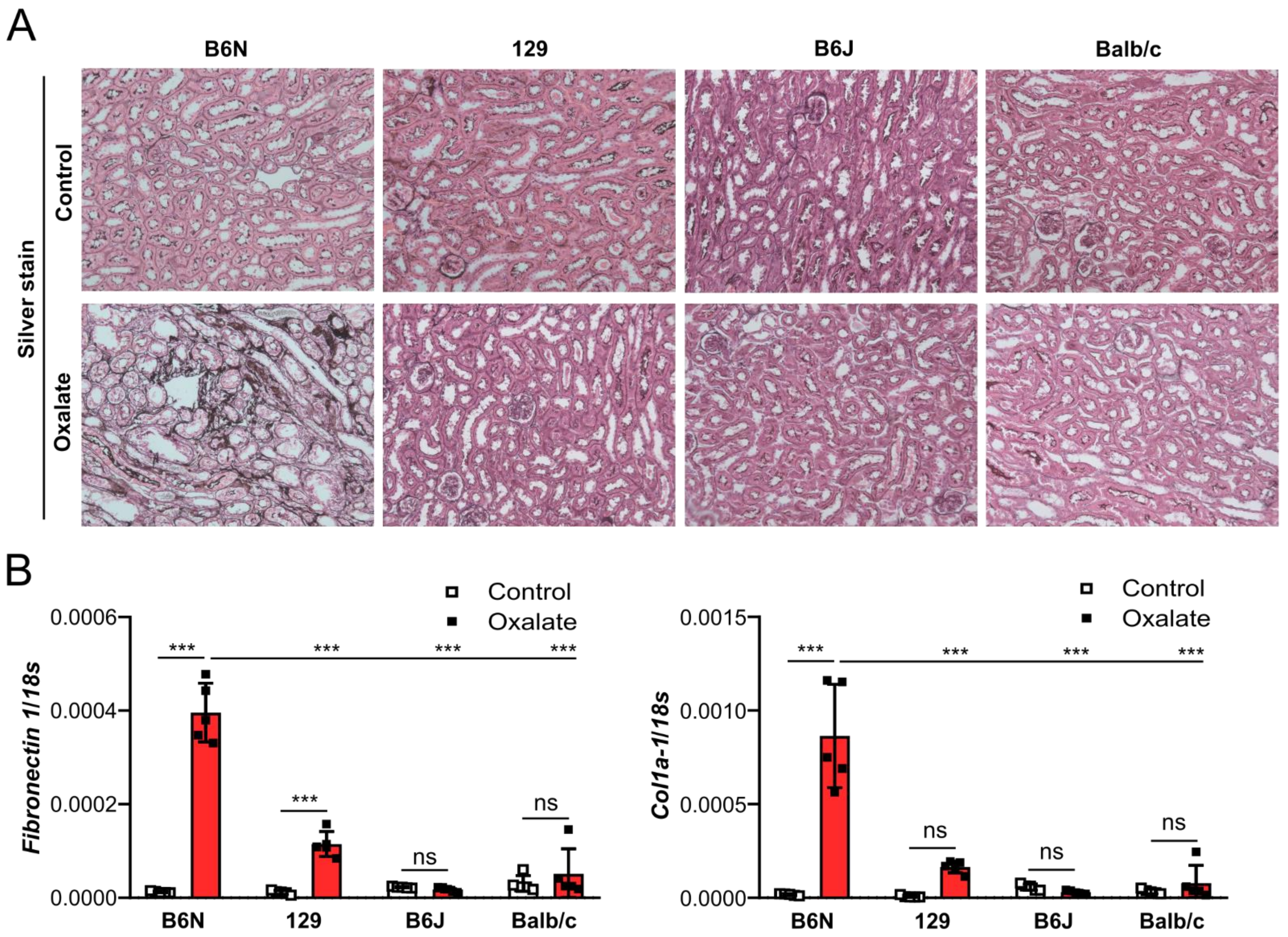

Supplementary Figure S2. B6N but not 129, B6J and Balb/c mice develop fibrosis upon feeding an oxalate-rich diet. B6N, 129, B6J and Balb/c mice were either placed on a control diet or an oxalate-rich diet for 14 days. (A) Silver staining to illustrate interstitial fibrosis in kidney sections of all four inbred mouse strains. Original magnification x200. (B) Gene expression of the fibrosis marker Fibronectin 1 and collagen (col)1α1 in kidney tissue determined by RT-PCR. Data are mean ± SD from 5 mice per group. *** p<0.001; ns, not significant.

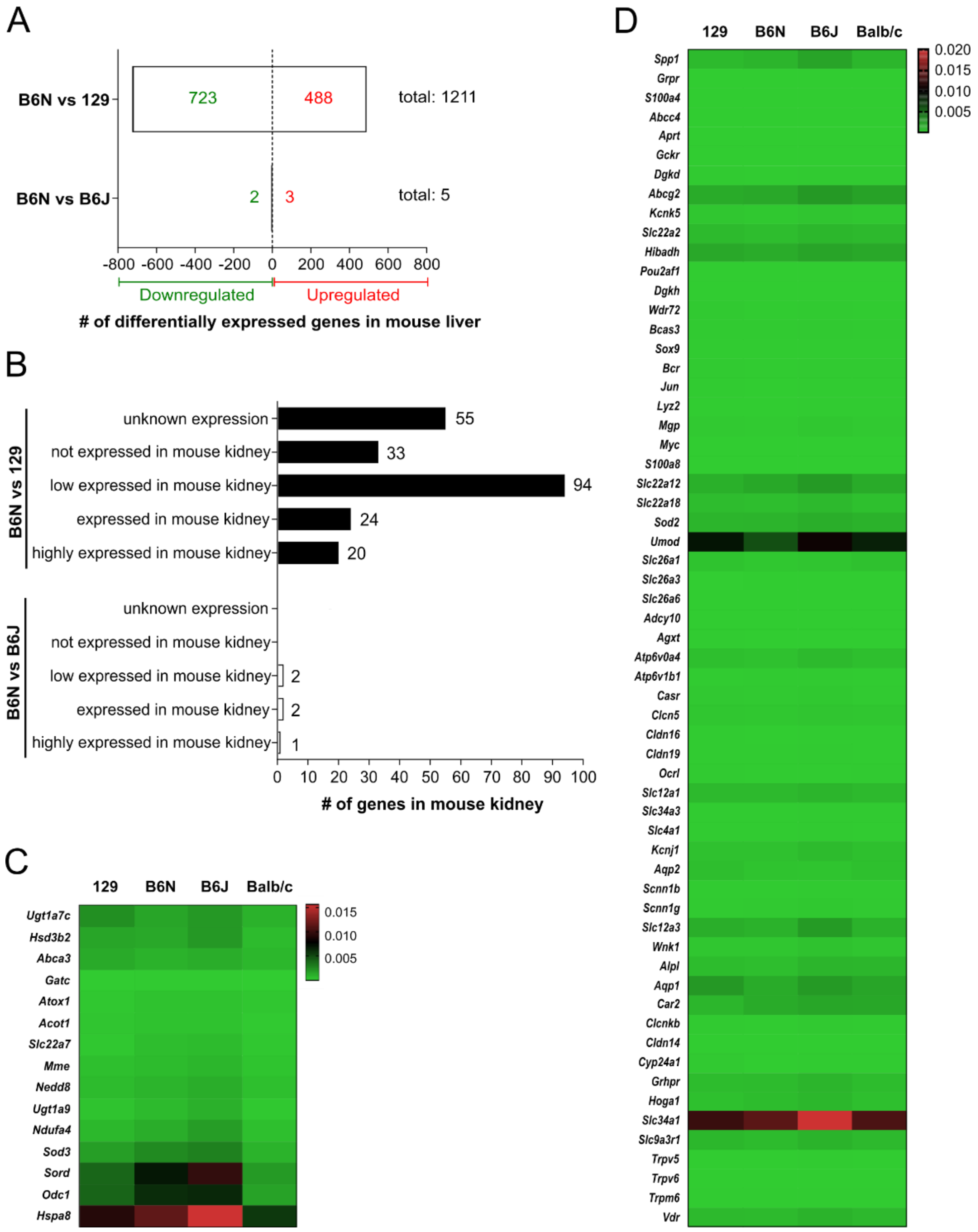

Supplementary Figure S3. Genetic variations in liver and kidney between B6N, 129, B6J and Balb/c mice. (A) Liver gene array data from healthy B6N, 129 and B6J mice (GSE43106). (B) Number of genes that are not, low, medium or highly expressed in the mouse kidney or with unknown expression according to the NCBI gene database (https://www.ncbi.nlm.nih.gov/gene). (C and D) Heat maps illustrating intrarenal mRNA expression levels of genes (ct values) identified via the NCBI gene database (C, 15 genes) and genes related to kidney stone disease (D, 64 genes) from healthy B6N, B6J, 129 and Balb/c mice determined by RT-PCR. Data are mean ± SD from 5 mice per group.

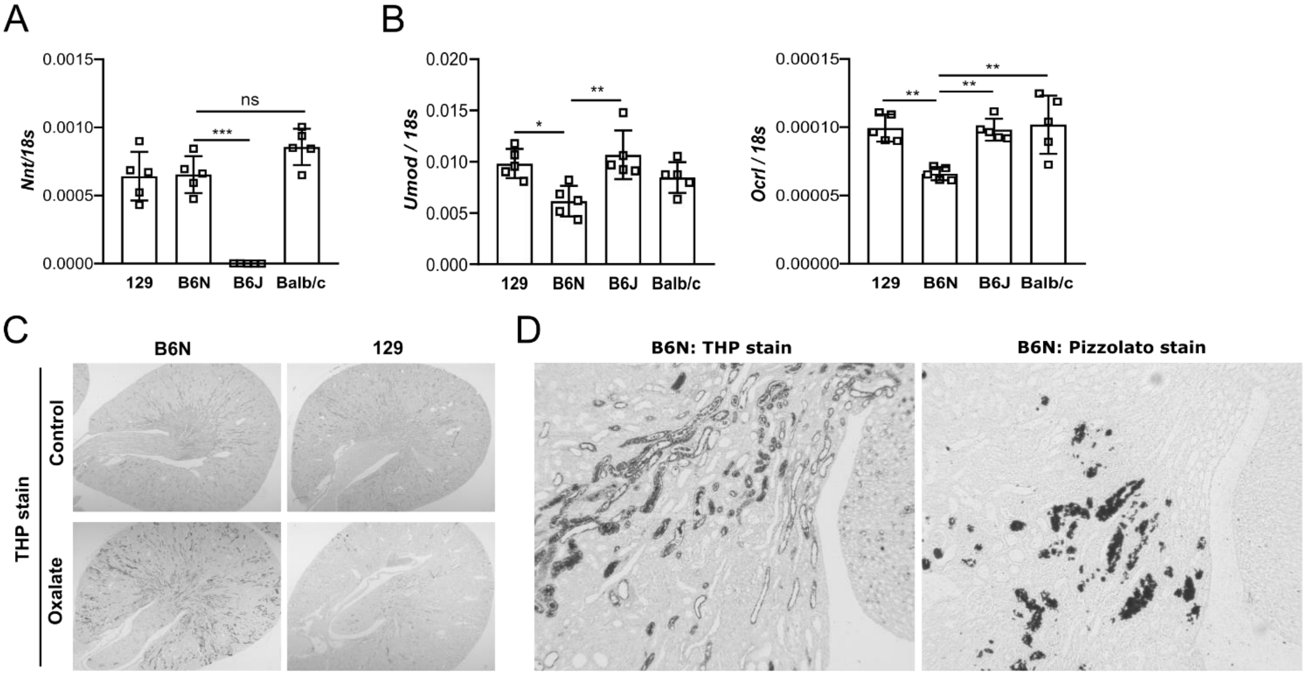

Supplementary Figure S4. Tamm-Horsfall Protein expression increases after feeding B6N mice an oxalate-rich diet. (A and B) Intrarenal mRNA expression levels of *Nnt* (A), *Umod* and *Ocrl* (B) in healthy B6N, 129, B6J and Balb/c mice determined by RT-PCR (one-way ANOVA). Data are mean ± SD from 5 mice per group. * p<0.05; ** p<0.01; *** p<0.001; ns indicates not significant. (C) Immunohistochemistry staining of kidney sections for Tamm-Horsfall Protein (THP) in B6N and 129 mice fed either a control or oxalate-rich diet for 14 days. Original magnification ×25. (D) THP and Pizzolato staining of kidney sections from B6N mice fed an oxalate-rich diet for 14 days. Original magnification ×400.

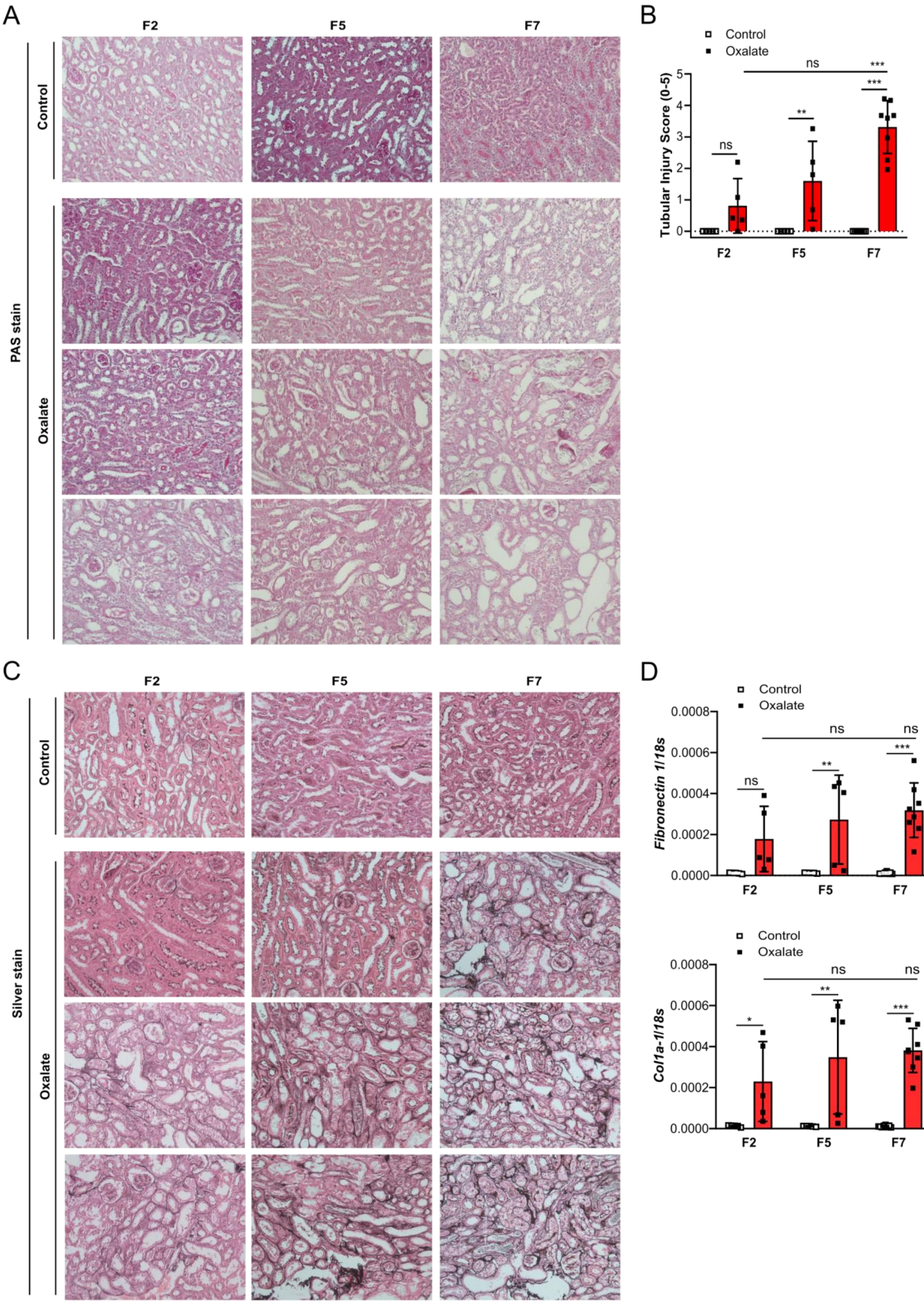
A
F2
F5
F7
Control
Oxalate
PAS stain
B
Control
Oxalate
ns
***
**
Tubular Injury Score (0-5)
0
1
2
3
4
5
F2
F5
F7
C
Silver stain
D
Fibronectin 1/18s
0.0000
0.0002
0.0004
0.0006
0.0008
*
Col1a-1/18s

Supplementary Figure S5. Backcrossing of 129 mice on a B6N background induced CaOx-crystal-related tubular injury and fibrosis. 129/B6N mice were generated by breeding 129 with B6N mice for up to 7 generations (F2, F5 and F7). 129/B6N F2, F5 and F7 mice were either placed on a control diet or oxalate-rich diet for 14 days. (A and B) Images of PAS stained kidney section (A) and quantification of the tubular injury score (B). Original magnification x200. (C) Silver stain of kidney sections illustrates interstitial fibrosis. Original magnification x200. (D) Intrarenal mRNA expression levels of *Fibronectin 1* and *Col1α1* determined by RT-PCR. Data are mean ± SD from 5 to 8 mice per group. * $p<0.05$; ** $p<0.01$; *** $p<0.001$; ns, not significant.

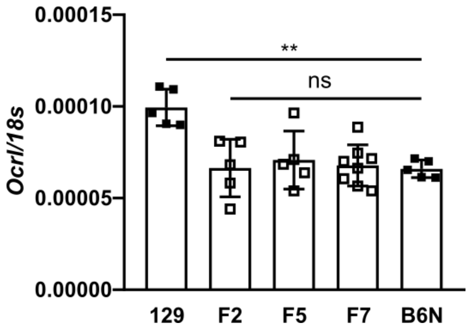

Supplementary Figure S6. Intrarenal mRNA expression levels of *Ocrl* in healthy 129, 129/B6N generation F2, F5 and F7 as well as B6N mice determined by RT-PCR (one-way ANOVA). Data are mean ± SD from 5 mice per group. * p<0.05; ** p<0.01; *** p<0.001; ns indicates not significant.

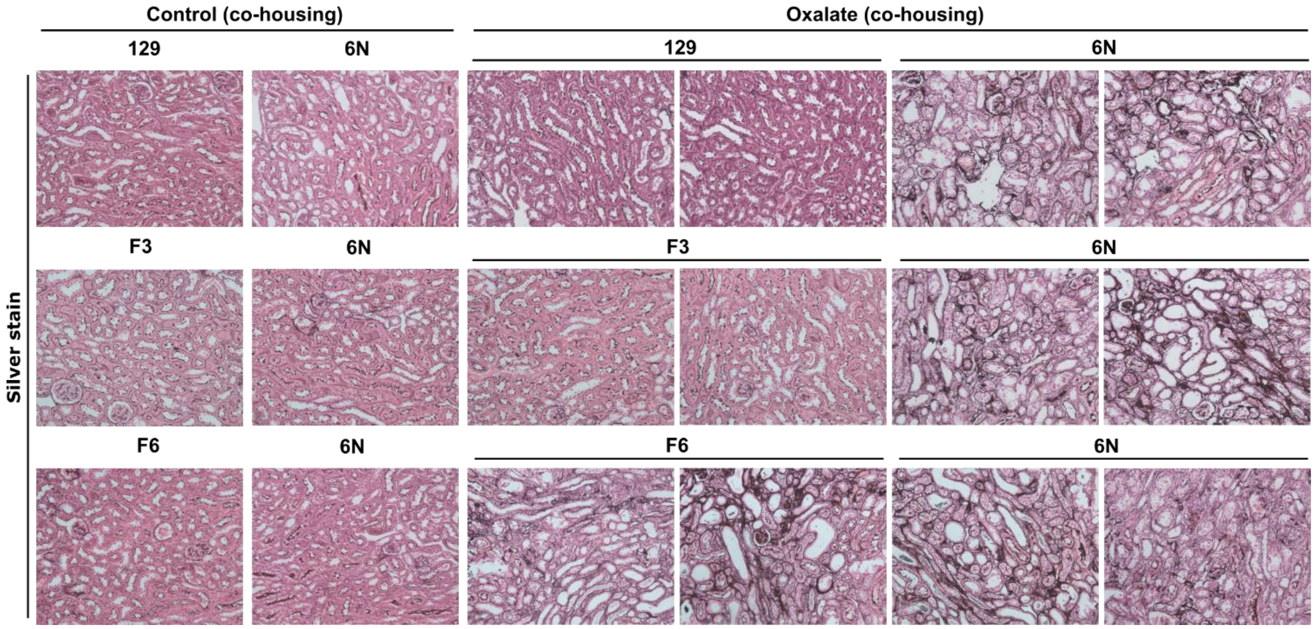

Supplementary Figure S7. Co-housing does not drive interstitial fibrosis in 129 mice on a B6N background. Three week-old B6N mice were co-housed either with 129 mice or B6N/129 F3 and F6 mice for 4 weeks, and then placed on a control diet or oxalate-rich diet for 14 days. Silver staining to illustrate interstitial fibrosis in kidney sections of all four mouse strains. Original magnification x200.

1.2 Supplementary Tables

Supplementary Table 1. Murine primer sequences

Mouse Gene	Primer Sequence
18s	Forward 5’- GCAATTATTCCCCATGAACG -3’<br>Reverse 5’- AGGGCCTCACTAAACCATCC -3’
KIM-1	Forward 5’- TCAGCTCGGGAATGCACAA -3’<br>Reverse 5’- TGGTTGCCTTCCGTGTCTCT -3’
Col1a1	Forward 5’- ACATGTTCAGCTTTGTGGACC -3’<br>Reverse 5’- TAGGCCATTGTGTATGCAGC -3’
Fibronectin 1	Forward 5’- GGAGTGGCACTGTCAACCTC -3’<br>Reverse 5’- ACTGGATGGGGTGGGAAT -3’
CD44	Forward 5’- AGCGGCAGGTTACATTCAAA -3’<br>Reverse 5’- CAAGTTTTGGTGGCACACAG -3’
Annexin II	Forward 5’- GCACATTGCTGCGGTTTGTCAG -3’<br>Reverse 5’- CACCAACTTCGATGCTGAGAGG -3’
Tnfα	Forward 5‘-CCACCACGCTCTTCTGTCTAC-3‘<br>Reverse 5‘-AGGGTCTGGGCCATAGAACT-3‘
Il6	Forward 5′-TGATGCACTTGCAGAAAACA-3′<br>Reverse 5′-ACCAGAGGAAATTTTCAATAGGC-3′
Spp1	Forward 5’- GCTTGGCTTATGGACTGAGGTC -3’<br>Reverse 5’- CCTTAGACTCACCGCTCTTCATG -3’
Grpr	Forward 5’- GAGTGCCTACAATCTTCCCGTG -3’<br>Reverse 5’- CAGAGTAGTGGTAGGAACGGTAC -3’
S100a4	Forward 5’- AGCTCAAGGAGCTACTGACCAG -3’<br>Reverse 5’- GCTGTCCAAGTTGCTCATCACC -3’
Abcc4	Forward 5’- CACACGATGCAGCTACCAGTGA -3’<br>Reverse 5’- GGTCATCCAGAGAAGCCTCCAT -3’
Aprt	Forward 5’- GTCATTGTGGATGACCTCCTGG -3’<br>Reverse 5’- TGGTATAGGTCCTAGCCTCTCC -3’
Gckr	Forward 5’- AGACTGTGGACCAGGGTGTTGT -3’<br>Reverse 5’- GCTGATGCCAACTTGCTTCGTC -3’
Dgkd	Forward 5’- GCCTATGAGAAGACGACAGAGAG -3’<br>Reverse 5’- GAGGATGAAGCCTGAGACTCGT -3’
Abcg2	Forward 5’- CAGTTCTCAGCAGCTCTTCGAC -3’<br>Reverse 5’- TCCTCCAGAGATGCCACGGATA -3’
Kcnk5	Forward 5’- CATCTCCACCATTGGCTTTGGG -3’<br>Reverse 5’- CCACAAACATGCTCACCTTCCAG -3’
Slc22a2	Forward 5’- CGGAGTCTCCAAGATGGTTGATC -3’<br>Reverse 5’- CCAGTATCCTCATCTGCCGTCA -3’
Hibadh	Forward 5’- ATGCTGGTCCAGCGACACTTAC -3’<br>Reverse 5’- GCAGAGTCTTGAGCCAATCCCA -3’
Pou2af1	Forward 5’- GTGTTCGAGTCAAGGAGCCAGT -3’<br>Reverse 5’- GCAGAAACCTCCATGTCAAGGC -3’
Dgkh	Forward 5’- CCAAAGTGGAGAAGGCACAGGA -3’<br>Reverse 5’- GCAAGGCTTGTAGCAACTGCTC -3’
Wdr72	Forward 5’- GGAAGCACCTTTTCCCTGTGAG -3’

	Reverse 5'- GCTCTTTCTCCTGTCTCATGCC -3'
Bcas3	Forward 5'- GACTTCCAGGTCGTGGTTTGCA -3'<br>Reverse 5'- ATGTGCTCCACTAAGGTCCCGT -3'
Sox9	Forward 5'- CACACGTCAAGCGACCCATGAA -3'<br>Reverse 5'- TCTTCTCGCTCTCGTTCAGCAG -3'
Bcr	Forward 5'- CTGCTCTACAAACCTGTGGACC -3'<br>Reverse 5'- TCTCCTCGTTGATGCTGGACAG-3'
Jun	Forward 5'- CAGTCCAGCAATGGGCACATCA -3'<br>Reverse 5'- GGAAGCGTGTTCTGGCTATGCA -3'
Lyz2	Forward 5'- TGCCAGAACTCTGAAAAGGAATGG -3'<br>Reverse 5'- CAGTGCTTTGGTCTCCACGGTT -3'
Mgp	Forward 5'- AGGAACGCAACAAGCCTGCCTA -3'<br>Reverse 5'- CTGCCTGAAGTAGCGGTTGTAG -3'
Myc	Forward 5'- TCGCTGCTGTCCTCCGAGTCC -3'<br>Reverse 5'- GGTTTGCCTCTTCTCCACAGAC -3'
S100a8	Forward 5'- CAAGGAAATCACCATGCCCTCTA -3'<br>Reverse 5'- ACCATCGCAAGGAACTCCTCGA -3'
Slc22a12	Forward 5'- GGAGGAACCAAGCAGGGACAAA -3'<br>Reverse 5'- CCGTAGAAGGTGAAGCCAAAGG -3'
Slc22a18	Forward 5'- GTCCTCAGCTTTACCTGTGTCC -3'<br>Reverse 5'- CTTGACCAGGAATACAGGCAGC -3'
Sod2	Forward 5'- TAACGCGCAGATCATGCAGCTG -3'<br>Reverse 5'- AGGCTGAAGAGCGACCTGAGTT -3'
Umod	Forward 5'- TGACCAACTGCTATGCCACACC -3'<br>Reverse 5'- GCCTGAGATGACTCGCCATTCT -3'
Slc26a1	Forward 5'- GATCATTGGGCTACAGGACTGC -3'<br>Reverse 5'- GAGAGATAGGTAGACACGAAGCC -3'
Slc26a3	Forward 5'- CAGTTTGCTGAAATAGGCAGATTG -3'<br>Reverse 5'- GCCTAATCCGAGTCCAAGGACA -3'
Slc26a6	Forward 5'- TACCGTGTGGACAGTAACCAGG -3'<br>Reverse 5'- CCTGTACCAAGCTCCGAGACAT -3'
Adcy10	Forward 5'- CTTGTGAGTGGTGTCAGGCTGA -3'<br>Reverse 5'- CAAACAGCAGCTCCGTGGTGAA -3'
Agxt	Forward 5'- CGGTATTGCTGTTCCTGGTCCA -3'<br>Reverse 5'- AGTCCACCAGGAGTAGGCACTG -3'
Atp6v0a4	Forward 5'- CAGTCATCCTGGGAATCGCTCA -3'<br>Reverse 5'- CCAGGTATCCAAACAGACTCAGC -3'
Atp6v1b1	Forward 5'- CCAGAAAGGACCACGGAGATGT -3'<br>Reverse 5'- AGGAACTCCAGGTAGAGCAGGT -3'
Casr	Forward 5'- AGGAAGGGCATCATTGAGGGAG -3'<br>Reverse 5'- CTCATTGGACCAGAAGTCATCCG -3'
Clcn5	Forward 5'- GCACCTTATGCCTGTGGTTCTG -3'<br>Reverse 5'- CAGGCTCAAGCCAGATGACACC -3'
Cldn16	Forward 5'- CAGACTGTTGGATGGTGAACGC -3'<br>Reverse 5'- TGGAGTCGTACTCATCGCAGGT -3'
Cldn19	Forward 5'- GCACTCGGGTTGGAGACAGTAA -3'

	Reverse 5’- GGTAGCATACCAGGAGACAGCA -3’
Ocrl	Forward 5’- AACCTTGGCTCGACTGTGATCC -3’<br>Reverse 5’- CTTTCCACAGCCAAGGACCACT -3’
Slc12a1	Forward 5’- CCAATGCAGTGGCTGTCGCTAT -3’<br>Reverse 5’- AATCACCACGGTGATGGAACCG -3’
Slc34a3	Forward 5’- GCGGTATTACCAGCAACACCAC -3’<br>Reverse 5’- TGTCCTCCTCTGGAGATGCTGA -3’
Slc4a1	Forward 5’- CTTGTGCTAGGCTTCTCAGGAC -3’<br>Reverse 5’- CCACCAACATCACGAGCAGGAT -3’
Kcnj1	Forward 5’- CTCTGTCTCCTCATCCGAGTAG -3’<br>Reverse 5’- TGGTCTCTCCTTCAGGTGTGATG -3’
Aqp2	Forward 5’- GCCATCCTCCATGAGATTACCC -3’<br>Reverse 5’- CGCTCATCAGTGGAGGCAAAGA -3’
Scnn1b	Forward 5’- ATGAACGGCTCCGATGTTGCCA -3’<br>Reverse 5’- CTCTCCTTCAGGCAGTGGGTAT -3’
Scnn1g	Forward 5’- AGAACTGCTGGTGACCTGCTTC -3’<br>Reverse 5’- GTGCTGAGGATGGTGGCGTTTT -3’
Slc12a3	Forward 5’- CTCCTACCTTGCCATCTCAGCT -3’<br>Reverse 5’- GGAGCATTCTGTGAAGTTCCAGC -3’
Wnk1	Forward 5’- CTACATCCTCCCAGCAAGCAGT -3’<br>Reverse 5’- CAGAGGTAACCAATGGAACAGGC -3’
Alpl	Forward 5’- CCAGAAAGACACCTTGACTGTGG -3’<br>Reverse 5’- TCTTGTCCGTGTCGCTCACCAT -3’
Aqp1	Forward 5’- CTTGCCATTGGCTTGTCTGTGG -3’<br>Reverse 5’- CCAGTGGTTTGAGAAGTTGCGG -3’
Car2	Forward 5’- CTCTGCTGGAATGTGTGACCTG -3’<br>Reverse 5’- CCAGTTGTCCACCATCGCTTCT -3’
Clcnkb	Forward 5’- CACCTCCATCTACAAGACCAGC -3’<br>Reverse 5’- GAAAGTCCGCTGGCTGTAGTTG -3’
Cldn14	Forward 5’- ACGCTCATCACCACCATTCTGC -3’<br>Reverse 5’- CTGACACTGGTAGATGCCTGTG -3’
Cyp24a1	Forward 5’- GCTCCTTCAAAAGGACACAGAGG -3’<br>Reverse 5’- CGCTTGCCACACTTTGGTGTTG -3’
Grhpr	Forward 5’- AGCCAACCTCAGAGTCATCAGC -3’<br>Reverse 5’- AGTGGCATCTGTCAGGACACCT -3’
Hoga1	Forward 5’- CTGCCCTCATTCACCACTACAC -3’<br>Reverse 5’- ACCACTGTCCTTCAAGCCGATG -3’
Slc34a1	Forward 5’- GGCTCCAACATTGGCACTACCA -3’<br>Reverse 5’- ACCACAGTAGGATGCCCGAGAT -3’
Slc9a3r1	Forward 5’- ATCTGCCTCCAGCGATACCAGT -3’<br>Reverse 5’- AGCCAAGGAGATGTTGAGGTCC -3’
Trpv5	Forward 5’- AGAGTTGGTGCCTCTCGCTACT -3’<br>Reverse 5’- CGCCATTCACATTGGTAAGCCTC -3’
Trpv6	Forward 5’- GAAGCGGAAACACATCCAGTGG -3’<br>Reverse 5’- ACAGGTGTCTGGTCCAGGATCT -3’
Trpm6	Forward 5’- CGTGACATCGTCTTTGAGCCGT -3’<br>Reverse 5’- GGTAGACAGCTTGCAGGAATGG -3’

Vdr	Forward 5’- GCTCAAACGCTGCGTGGACATT -3’<br>Reverse 5’- GGATGGCGATAATGTGCTGTTGC -3’
Nnt	Forward 5’- TCTCCACTCACCGCTGATGTCT -3’<br>Reverse 5’- AGCAGCAAGACTCTGAGAAGTTG -3’
Ugt1a7c	Forward 5’- GCTGAGGCTTTGGGCAGAATTC -3’<br>Reverse 5’- GTGATGAATGCCCGAGTCTTTGG -3’
Hsd3b2	Forward 5’- ATCAGGGTCCTGGACAAGGTCT -3’<br>Reverse 5’- TGGCAAGCTCTCCTCAGGTACT -3’
Abca3	Forward 5’- CTTCATGGACGAAGCTGACCTG -3’<br>Reverse 5’- GTGCGGTTCTTTTACCAGCGTC -3’
Gatc	Forward 5’- CTGACAATGTTGCAGAAGGCAGC -3’<br>Reverse 5’- GTTTACCATGTCTGGCAAGGAAAT -3’
Atox1	Forward 5’- GTGTGCCGCGTCAGTCAT -3’<br>Reverse 5’- ACTCCTCCCAGCTTGTTGAG -3’
Acot1	Forward 5’- AAGAAGCCGTGAACTACCTGCG -3’<br>Reverse 5’- TGTGATGCCCTTCAGGAAGGAG -3’
Slc22a7	Forward 5’- ACTGCTCTGACCTTCGGCATCA -3’<br>Reverse 5’- CAGACGTGAACAGGTAGGCTGT -3’
Mme	Forward 5’- CAGCCGAAACTACAAGGAGTCC -3’<br>Reverse 5’- CATAAAGCCTCCCCACAGCATTC -3’
Nedd8	Forward 5’- GGAGAAGCAGCACTCTAGCC -3’<br>Reverse 5’- CTCCACCTTGTCTGTGGGTT -3’
Ugt1a9	Forward 5’- TTGGTGGGATCAACTGCCTCCA -3’<br>Reverse 5’- CGGAATCTCTGAGACCATGGATC -3’
Ndufa4	Forward 5’- AGCAGCACTGTATGTGATGCGC -3’<br>Reverse 5’- TGTAGTCCACATTCACAGAGTAGA -3’
Sod3	Forward 5’- GACCTGGTTGAGAAGATAGGCG -3’<br>Reverse 5’- TGGCTGATGGTTGTACCCTGCA -3’
Sord	Forward 5’- GGATGGTCACTTTGCTTGTGGC -3’<br>Reverse 5’- GGTCTCTTTGCCAACCTGGATG -3’
Odc1	Forward 5’- ACATCCAAAGGCAAAGTTGG -3’<br>Reverse 5’- AGCCTGCTGGTTTTGAGTGT -3’
Hspa8	Forward 5’- CCGATGAAGCTGTTGCCTATGG -3’<br>Reverse 5’- CCAAGGGAAAGAGGAGTGACATC -3’
